# Characterizing the Diversity of Layer 2/3 Human Neocortical Neurons in Pediatric Epilepsy

**DOI:** 10.1523/ENEURO.0247-24.2025

**Published:** 2025-05-02

**Authors:** J. Keenan Kushner, Paige B. Hoffman, Christine R. Brzezinski, Matthew N. Svalina, Brent R. O’Neill, Todd C. Hankinson, Charles C. Wilkinson, Michael H. Handler, Serapio M. Baca, Molly M. Huntsman, Allyson L. Alexander

**Affiliations:** ^1^Neuroscience Graduate Program, University of Colorado | Anschutz Medical Campus, Aurora, Colorado 80045; ^2^Department of Pharmaceutical Sciences, Skaggs School of Pharmacy and Pharmaceutical Sciences, University of Colorado | Anschutz Medical Campus, Aurora, Colorado 80045; ^3^Department of Neurosurgery, School of Medicine, University of Colorado | Anschutz Medical Campus, Aurora, Colorado 80045; ^4^Medical Scientist Training Program, University of Colorado | Anschutz Medical Campus, Aurora, Colorado 80045; ^5^Department of Cell and Developmental Biology, University of Colorado | Anschutz Medical Campus, Aurora, Colorado 80045; ^6^Department of Pharmacology, University of Virginia, Charlottesville, Virginia 22903; ^7^Department of Pediatrics, School of Medicine, University of Colorado | Anschutz Medical Campus, Aurora, Colorado 80045

**Keywords:** DNET, focal cortical dysplasia, gliosis, layer 2/3 pyramidal neurons, intrinsic properties, tuberous sclerosis

## Abstract

Childhood epilepsy is a common and devastating condition, for which many children still do not have adequate treatment. Some children with drug-resistant epilepsy require surgical excision of epileptogenic brain tissue for seizure control, affording the opportunity to study this tissue ex vivo to interrogate human epileptic neurons for potentially hyperexcitable perturbations in intrinsic electrophysiological properties. In this study, we characterized the diversity of layer L2/3 (L2/3) pyramidal neurons (PNs) in ex vivo brain slices from pediatric patients with epilepsy. We found a remarkable diversity in the firing properties of epileptic L2/3 PNs: five distinct subpopulations were identified. Additionally, we investigated whether the etiology of epilepsy influenced the intrinsic neuronal properties of L2/3 PNs when comparing tissue from patients with epilepsy due to malformations of cortical development (MCDs), other forms of epilepsy (OEs), or with deep-seated tumors. When comparing epileptic with control L2/3 PNs, we observed a decrease in voltage sag and lower maximum firing rates. Moreover, we found that MCD and OE L2/3 PNs were mostly similar indicating that epilepsy etiology may not outweigh the influences of epileptiform activity on L2/3 PN physiology. Lastly, we show that the proconvulsant drug, 4-aminopyridine (4-AP), leads to increased AP half-width, reduced firing rate accommodation, and slower AHPs. These changes imply that 4-AP induces an increase in [K^+^]_o_ and a resultant increase in AP duration, leading to the release of more excitatory neurotransmitters per action potential, thereby promoting network hyperexcitability.

## Significance Statement

This study characterizes the diversity of L2/3 PNs within human epileptic loci ex vivo. We identify significant differences in L2/3 PN intrinsic properties between epileptic subtypes and control L2/3 PNs, and these differences promote increased synaptic summation and neurotransmission. Furthermore, we document that AHP kinetics do not dictate epileptic L2/3 PN firing rates. We also find that L2/3 PNs demonstrate commensurate properties regardless of the etiology of epilepsy. Finally, we document the effects of the convulsant drug 4-AP, on epileptic L2/3 PN intrinsic properties. This study contributes to the understanding of human neocortical epilepsy, the effects on neuron subtypes using pro-ictal drugs to generate seizures ex vivo, and the neuronal abnormalities associated with epileptogenesis.

## Introduction

Childhood epilepsy is a common disorder, with a reported prevalence ranging from 0.3 to 4.4% (Camfield and Camfield, 2015; Aaberg et al., 2017). Antiseizure medications (ASMs) remain the first-line treatment for children with epilepsy, yet approximately one-third of children develop drug-resistant epilepsy and continue to have seizures (Golyala and Kwan, 2017). Malformations of cortical development (MCDs), including focal cortical dysplasia (FCD), hemimegalencephaly (HMG), and tuberous sclerosis complex (TSC), are the most common causes of surgically treated epilepsy in the pediatric population (Blumcke et al., 2017). Although surgery is curative for many patients with MCDs, not all patients are eligible for surgery and 5 year seizure freedom rates after surgery are <70% (Lamberink et al., 2020). Thus, there remains a substantial proportion of patients who continue to have seizures despite optimal medical and surgical therapy.

Over the last few decades, many studies have characterized human epileptic cortical neurons and investigated the mechanisms that underlie interictal and ictal activity (Hwa et al., 1991; D'Antuono et al., 2004; Avoli et al., 2005; Cepeda et al., 2006; Kohling and Avoli, 2006; Jacobs et al., 2008; Huberfeld et al., 2011; Talos et al., 2012; Abdijadid et al., 2015; Blauwblomme et al., 2019; Levinson et al., 2020; Rossini et al., 2021). A common neocortical layer of focus is layer 2/3 (L2/3), which controls the gain of cortical output (Quiquempoix et al., 2018). Prior pediatric MCD ex vivo studies have demonstrated that L2/3 generates ictal discharges, abnormal bursting activity, and pathogenic high-frequency oscillations (HFOs) and has shown that these epileptiform signatures are significantly regulated by NMDA receptors, GABAergic neurotransmission, and dopaminergic modulation (Wuarin et al., 1990; Cepeda et al., 1992; Mattia et al., 1995; Avoli et al., 1999; D'Antuono et al., 2004; Calcagnotto et al., 2005; Blauwblomme et al., 2019; Levinson et al., 2020). However, few of these prior studies include nonepileptic control patients or compared results between epileptic subtypes.

For many MCDs, genetic alterations in the mammalian target of rapamycin (mTOR) underlie the developmental malformation and presumably drive epileptogenesis (Jozwiak et al., 2006; Cepeda et al., 2012; O'Dell et al., 2012; Heinemann and Staley, 2014; Lipton and Sahin, 2014; Crino, 2015; Guerrini et al., 2015; D'Gama et al., 2017; Iffland and Crino, 2017; Nakagawa et al., 2017; Curatolo et al., 2018; Park et al., 2018; Wu et al., 2021). Other common pathologies leading to surgically treatable focal-onset epilepsy include perinatal stroke, often pathologically characterized by gliosis or encephalomalacia, and idiopathic forms of epilepsy, often characterized as low-grade tumors. Based on the mechanistic, developmental, and cellular differences between MCD epilepsies and other epilepsies, we hypothesized that L2/3 PNs of these subclasses differ in their intrinsic properties.

Despite the intrinsic hyperexcitability of human brain tissue resected during epilepsy surgery, ex vivo ictal activity is only induced via pharmacologic manipulations or alterations in the concentration of extracellular cations (Chang et al., 2019). One reproducible method for the generation of epileptiform activity in rodent or human tissue is wash on of 4-aminopyridine (4-AP), an A-type K^+^ channel blocker (Chesnut and Swann, 1988; Avoli and Jefferys, 2016; Chang et al., 2019). Addition of 4-AP has been shown to increase action potential (AP) duration, elicit a large increase in extracellular [K^+^] and intracellular [Ca^2+^]; generate a GABAergic, long-lasting depolarizing potential, reduce firing rate accommodation, and augment neurotransmitter release leading to interictal and ictal discharges (Lorenzon and Foehring, 1992; Kohling and Avoli, 2006; Wu et al., 2009; Abdijadid et al., 2015; Williams and Hablitz, 2015). Moreover, 4-AP allows for the examination of how voltage-gated K^+^ channels (members of the Kv1, Kv3, and Kv4 families) play a role in hyperexcitability, as 4-AP has no effect on Na^+^ or Ca^2+^ channels (Wulff and Zhorov, 2008). Although 4-AP can elicit ictal activity in brain slices from control and epileptic tissue, there has been little prior study of the differing effects of 4-AP on these respective tissue types. Therefore, we examined the effects of 4-AP on L2/3 PN intrinsic properties to gain a better understanding of how this potent convulsant elicits ictal activity ex vivo.

Here, we present a systematic analysis of the diversity of L2/3 PNs in human epileptic tissue. This study, to the best of our knowledge, is the first to characterize the diversity of L2/3 PNs from human epileptic foci including the differences in firing rate properties, passive membrane properties, and AP kinetics. In addition, it is the first to show differences in L2/3 PN intrinsic properties between MCD, other epilepsies, and control tissues and determines the direct effect of 4-AP on epileptic L2/3 PN intrinsic properties.

## Materials and Methods

### Acute human brain slice preparation for electrophysiology

Resected neocortical tissue was obtained from Children's Hospital Colorado. The decision to recommend epilepsy surgery is always made by a multidisciplinary team including board-certified physicians in pediatric epileptology, pediatric neurosurgery, pediatric neuroradiology, and a pediatric neuropsychologist specializing in patients with epilepsy. All patients, or their legal guardians, provided written informed consent for the study before surgery. If appropriate, the patient also provided assent. This process was performed in accordance with our institution's Institutional Review Board. For the present study, cortical tissue samples were collected from 33 patients in three experimental groups: MCD epilepsy, other epilepsy, and control. Control patients were patients with deep-seated neoplastic lesions, which required removal of normal (nonepileptogenic, non-neoplastic) superficial cortex to resect the tumor. No additional cortex was removed beyond that which was needed for resection of the deep-seated lesion. In this study, we used tissue resected from 28 epilepsy and five tumor control patients. Patients ranged from 1 to 21 years of age (mean, 9.9). Twenty were male and 13 were female. The brain samples analyzed in the present experiments were from the temporal (*n* = 15), frontal (*n* = 13), parietal (*n* = 4), or occipital (*n* = 1) neocortex. Patient information including age, sex, diagnosis, and experimental group is presented in Table 1. For all epilepsy patients, the resected neocortical tissue used for recordings was located in the epileptic focus.

Immediately following the surgical resection, resected tissue was submerged in 0–4°C carbogenated (95% O_2_–5% CO_2_) *N*-methyl-d-glucamine (NMDG) substituted artificial cerebrospinal fluid [ACSF; in mM: 92 NMDG, 2.5 KCl, 1.25 NaH_2_PO_4_, 30 NaHCO_3_, 20 4-(2-hydroxyethyl)-1-pip-erazineethanesulfonic acid (HEPES), 25 d-glucose, 2 thiourea, 5 Na-ascorbate, 3 Na-pyruvate, 0.5 CaCl_2_·4H_2_O, and 10 MgSO_4_·7H_2_O, pH adjusted to 7.3–7.4 with concentrated hydrochloric acid (HCl), osmolality ∼305 mOsm/kg]. The total duration from operating room to slicing was 15–20 min. The tissue was then placed in a carbogenated petri dish with 0–4°C carbogenated NMDG ACSF. Approximately 1 cm^3^ tissue blocks were prepared with a scalpel, if the specimen was larger than that size. The arachnoid, superficial blood vessels, and cauterized tissue were carefully removed from the specimen. No effort was made to remove the pia mater due to the risk of damaging the underlying gray matter. The ∼1 cm^3^ tissue block was then superglued on to a vibratome stage (Leica Biosystems) and immersed in 0–4°C carbogenated NMDG ACSF. Acute coronal slices (400 µm) were prepared by slicing perpendicular to the pial surface to preserve the laminated cortical structure as well as the primary dendrites of PNs. Slices were then incubated in carbogenated NMDG ACSF at ∼35°C for 12 min and were then transferred to a room temperate (∼23°C) carbogenated modified HEPES ACSF (in mM: 92 NaCl, 2.5 KCl, 1.2 NaH_2_PO_4_, 30 NaHCO_3_, 20 HEPES, 25 d-glucose, 2 thiourea, 5 Na-ascorbate, 3 Na-pyruvate, 2 CaCl_2_·4H_2_O, 2 MgSO_4_·7H_2_O, pH adjusted to 7.3–7.4 with HCl, osmolality ∼305 mOsm/kg). The slices remained in the ∼23°C modified HEPES ACSF for at least 30 min before being transferred to the recording chamber for electrophysiology experiments.

### Electrophysiology

For whole-cell patch-clamp recordings, slices were placed in a submerged slice chamber and perfused at a rate of 2 ml/min with heated (∼33°C) recording ACSF (in mM: 124 NaCl, 2.5 KCl, 1.2 NaH_2_PO_4_, 24 NaHCO_3_, 5 HEPES, 12.5 d-glucose, 2 CaCl_2_·4H_2_O, 2 MgSO_4_·7H_2_O, pH adjusted to 7.3–7.4 with HCl, osmolality ∼305 mOsm/kg). Slices were visualized using a moving stage microscope (Scientifica, SliceScope Pro 2000) equipped with 4× (0.10 NA) and a 40× water immersion objective lens (0.80 NA) objectives, differential interference contrast (DIC) optics, a SciCam Pro camera (Scientifica), and Micro-Manager 1.4 (Open Imaging). L2/3 PNs were visualized under DIC. Whole-cell patch-clamp recordings were performed with pipettes pulled to 3–6 MΩ and filled with the following internal solution (in mM: 135 potassium gluconate, 20 KCl, 10 HEPES,0.1 EGTA, 2 Mg-ATP, 0.3 Na_2_-GTP, pH adjusted to 7.3–7.4 with KOH, osmolality ∼290 mOsm/kg). For most recordings, 0.4% biocytin was added to the internal solution.

After achieving whole-cell configuration, L2/3 PNs were recorded from rest in current-clamp mode (*I*_hold_ = 0 pA). Following a 0.5 s baseline period, the holding current was linearly ramped from 0 to 1,000 pA over 2 s followed by a 2.5 s recovery period. Before initiation of the series of current injections, holding current was adjusted so that the resting membrane potential of neurons was approximately −60 mV. Each cell was subjected to two series of 600 ms square current injections: −100 to +100 pA at 10 pA intervals and −250 to +1,000 pA at 50 pA intervals. The following intrinsic properties were measured in each cell using the above current injections: resting membrane potential (mV), input resistance (MΩ), membrane decay (*τ*, ms), voltage sag (%), AP threshold, AP amplitude, AP half-width, magnitude of afterhyperpolarization (AHP, mV), AHP latency, ΔAHP, AP phase plot, AP broadening ratio, AP amplitude adaptation ratio, max firing rate (FR), FR ratio, instantaneous frequency, and frequency versus time. Extended Data Table 2-2 describes how each of the above parameters were calculated. In a subset of experiments, 100 µM of 4-aminopyridine (4-AP) was washed on to induce ictal activity. For the analysis of AHP kinetics in Figure 3, the AHPs were measured at rheobase and 2× rheobase.

Electrical recordings were acquired with a MultiClamp 700B amplifier and were sent through a Hum Bug Noise Eliminator (Quest Scientific) to then be converted to a digital signal with the Axon Digidata 1440A digitizer using pCLAMP 10.7 software (Molecular Devices). Access resistance was monitored throughout the experiments, and data were discarded if access resistance exceeded 25 MΩ. No junction potential compensation or series resistance compensation was performed. In current-clamp mode, compensation for voltage variations was achieved using a bridge balance circuit. Data were sampled at 10 kHz.

### Biocytin reconstructions

After the recordings were completed, the slice was then left to rest in the recording chamber for 4–5 min to ensure transport of biocytin to distal dendrites and axon processes (Swietek et al., 2016). The slice was then fixed in 4% PFA for 24–48 h. Biocytin was visualized with streptavidin conjugated to Alexa Fluor 488 or 594 at 1:1,000 (Jackson ImmunoResearch). Biocytin-filled neurons were visualized using a Zeiss Axio Imager M2 microscope. 3D slide scanning and neuron tracing was performed using Neurolucida software (MBF Bioscience).

### Statistical analyses

All data analysis was performed using MATLAB R2018a, GraphPad Prism 9.3.1, or Easy Electrophysiology v4.2.0 (http://www.easyelectrophysiology.com/). If data sets contained at least 10 data points, a D’Agostino and Pearson omnibus K2 normality test was performed to assess normality. For statistical tests between three or more groups with normally distributed data, 10 or more data points, and equal variance, an ANOVA test was performed. Equal variance was determined using the Brown–Forsythe test. If data was normally distributed but had unequal variance (Brown–Forsythe test *p* < 0.05), then a Welch's ANOVA test was performed. Following an ANOVA, a Tukey honestly significant difference (HSD) post hoc test was performed to find which group(s) differed. For statistical tests between three or more groups of non-normally distributed data, or data sets with <10 data points, a Kruskal–Wallis (KW) test was performed. Differences between groups were determined using Dunn's multiple-comparisons post hoc test and the *H* (degrees of freedom) statistic was checked against the critical chi-square value.

When comparing two groups with normally distributed data and standard deviations (SDs) between groups being <2×, an unpaired *t* test was applied. Two groups with non-normal data and/or data with SD being >2× between groups were compared using a Mann–Whitney *U* (MWU) test. For paired data, a paired *t* test was performed on normally distributed data with SD between groups being <2×, and a Wilcoxon matched-pairs signed rank test was performed on non-normal data and data where SD between groups was >2×.

All statistical tests were two-tailed. Unless otherwise stated, experimental numbers are reported as *n* = *x*, *y*, where *x* is the number of neurons and *y* is the number of patients. Statistical significance is notated in figures as the number of asterisks with the corresponding *p* values:*p < 0.05, **p < 0.01, ***p < 0.001, ****p < 0.0001. Data visualizations were created in MATLAB, GraphPad Prism, Adobe Illustrator, and OriginPro 2022. Data in tables and figure scatterplots are presented as mean ± SD, and figure data presented as line graphs are presented as mean ± SEM for ease of comparison. Frequency and FR ratio line graphs indicate smoothed data using the smoothing spline method in GraphPad Prism. Phase plots were created using the B-spline connect interpolation in OriginPro 2022.

### Code accessibility

The MATLAB scripts used for intrinsic property analysis are from a previous publication (Guthman et al., 2020).

## Results

### Subjects enrolled

**Figure 1.**
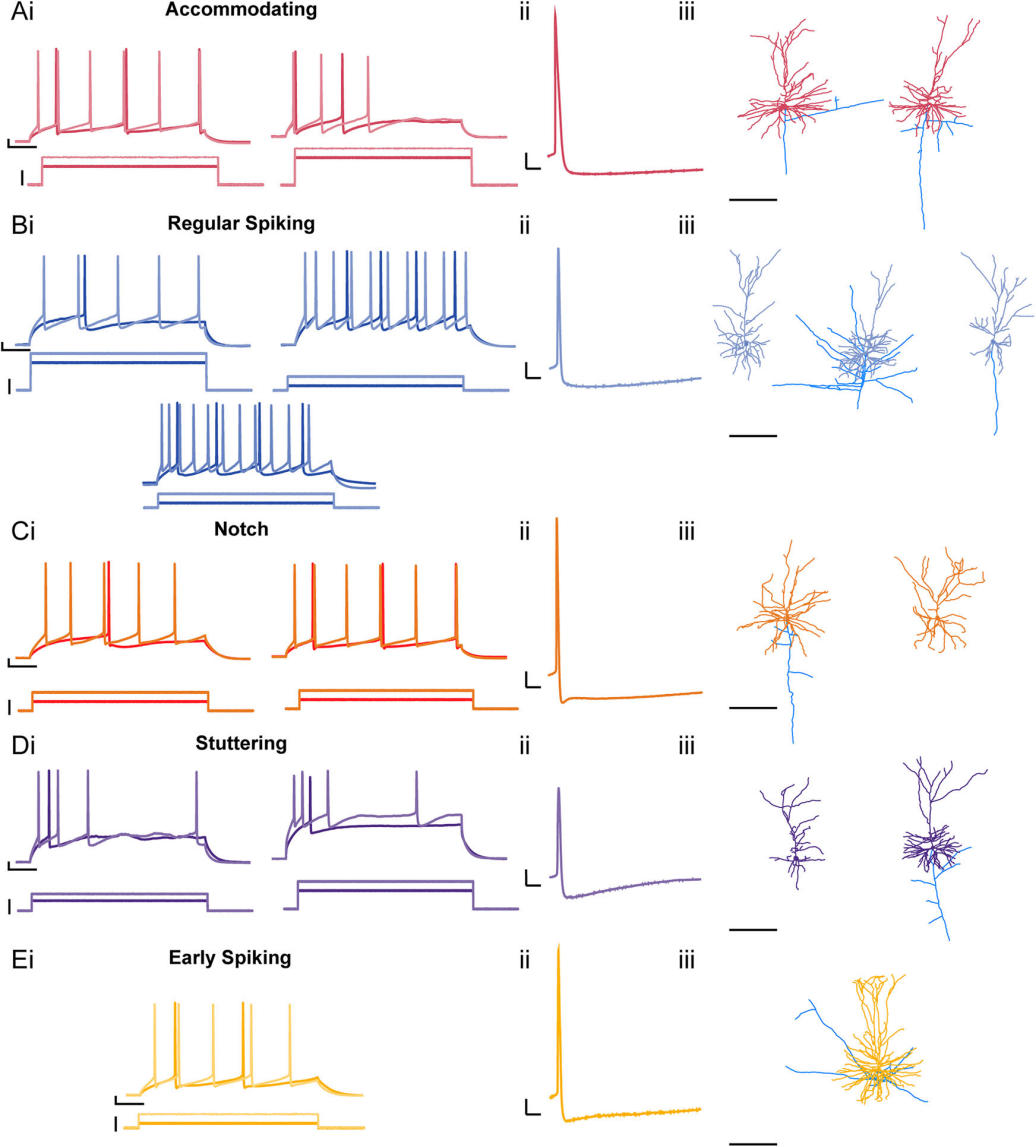
Putative L2/3 PNs from human epileptic foci have diverse morphology and spiking properties. ***Ai*–*Ei***, Depolarizing current steps at rheobase (pA; dark trace) and rheobase + 100 pA (2× rheobase, light trace) elicit action potentials (APs) of various shapes and frequencies. Each group was determined based on firing property and AHP shape. ***Aii*–*Eii***, Representative trace of each AP from neuron subtype (scale bars: 10 mV, 10 ms). ***Aiii*–*Eiii***, Corresponding 3D reconstruction of neuron morphology (*x*-axis scale bar: 200 µm). Each neuron's location in the representative cortical layers was determined by the respective distance of their soma from pial surface (dendrites: subtype color scheme, axon branches: blue).

**Figure 2.**
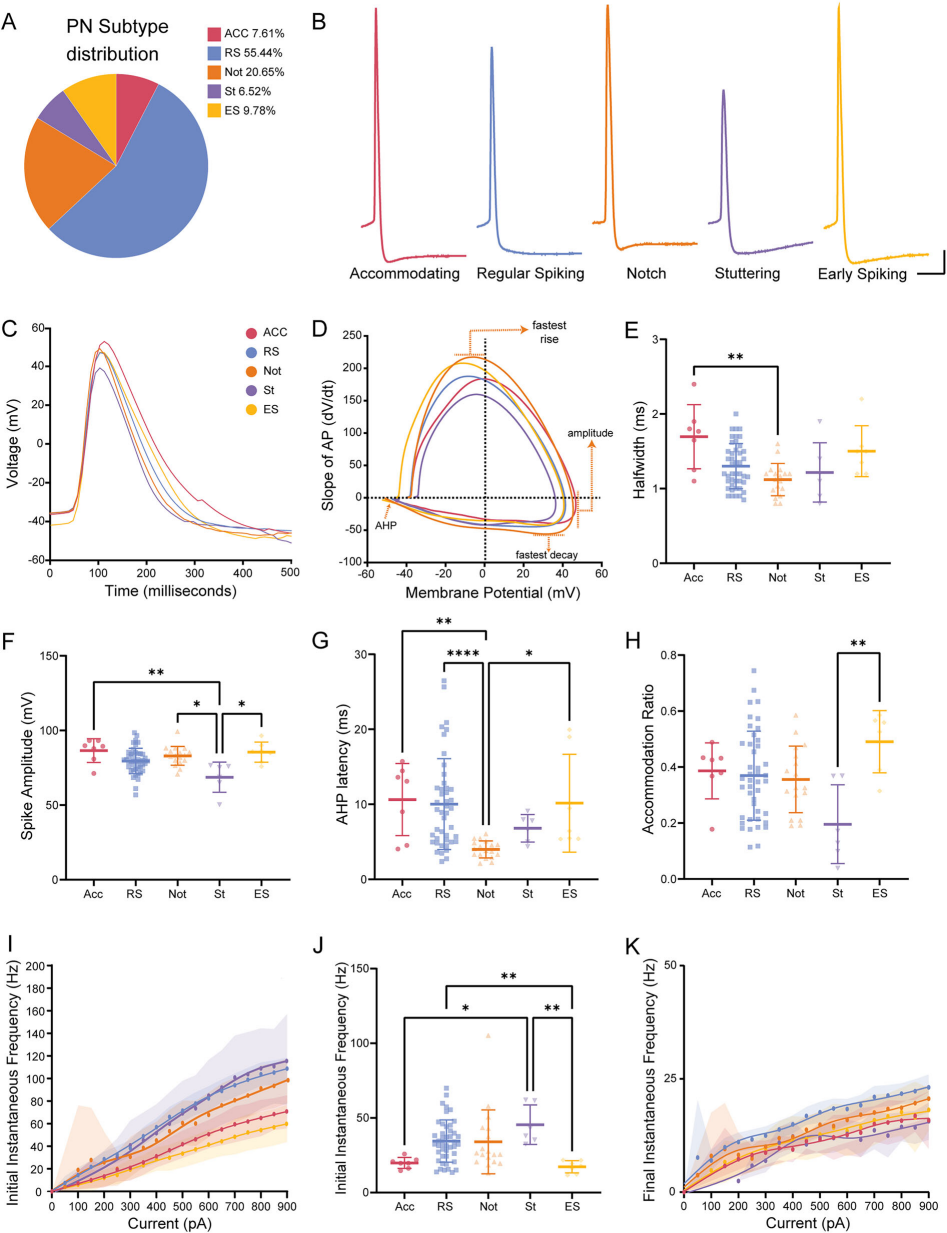
Putative L2/3 PN subtypes show subtle differences in AP kinetics and firing properties. ***A***, Neuron subtype percentage (%) of the entire population of recorded neurons (ACC, accommodating neurons; RS, regular spiking neurons; Not, notch neurons; ST, stuttering neurons; ES, early spiking neurons). ***B***, Representative traces of L2/3 PN subtype AP (scale bars: 10 mV, 10 ms). ***C***, Overlay of average AP of each neuron subtype. ***D***, Overlay of average phase plot (dV/dT vs voltage) indicating differences in subtype AP kinetics. ***E***, Notch neurons showed the shortest AP half-width with a significant difference compared with accommodating neurons. ***F***, Stutter neurons showed a significant reduction in AP spike amplitude. ***G***, Notch neurons have the shortest AHP latency with a significantly shorter AHP latency compared with RS and ACC neurons. ***H***, FR accommodation ratio of each neuron subtype taken at 2× rheobase indicated ES neurons had a significantly lower chance of accommodating compared with stutter neurons. ***I***, Initial instantaneous frequency ± SEM versus injected current (pA). ***J***, Initial instantaneous frequency was taken at the rheobase + 100 pA current step and indicated significant differences between subtype initial instantaneous firing frequency. ***K***, Final instantaneous frequency ± SEM versus injected current (pA). Scatterplots include mean values ± SD.

**Figure 3.**
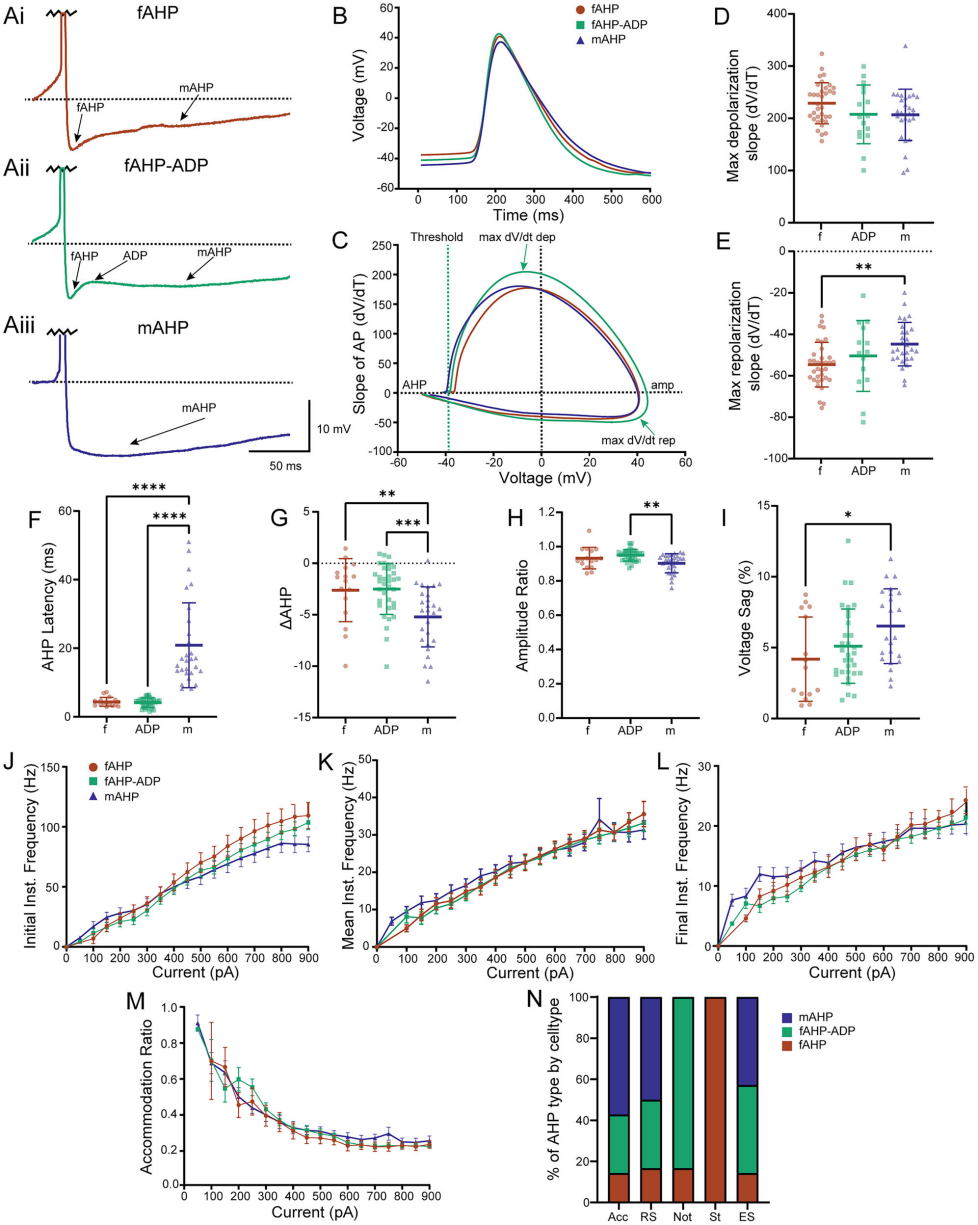
Epileptic L2/3 PNs with fAHP or mAHP do not differ in their firing rates or firing rate accommodation. ***A***, Representative traces of L2/3 PN AP shapes showing neurons with a (***Ai***) fAHP followed by mAHP, (***Aii***) fAHP followed by ADP and mAHP, and (***Aiii***) mAHP only. ***B***, Overlay of average AP of neuron split by AHP shape. ***C***, Overlay of average phase plots (dV/dT vs voltage) of neuron split by AHP shape. ***D***, There is no significant differences in the maximum depolarization slope of the AP (dV/dT) between subtypes. ***E***, mAHP PNs have a significantly slower repolarization slope (dV/dT) when compared with fAHP PNs. ***F***, AHP latency is shorter for fAHP and ADP PNs as expected compared with mAHP neurons. ***G***, fAHP and ADP PNs show a more positive ΔAHP. ***H***, mAHP PNs have more AP amplitude adaptation compared with ADP PNs. ***I***, mAHP PNs have higher overall voltage sag (%) compared with fAHP PNs. We observe no differences in ***J***, initial instantaneous frequency ± SEM versus injected current (pA); ***K***, mean instantaneous frequency ± SEM versus injected current (pA); ***L***, final instantaneous frequency ± SEM versus injected current (pA); or ***M***, FR accommodation ratio ± SEM versus injected current (pA). ***N***, Percentage (%) of AHP PN based on cell type characterized in Figure 2. f, fAHP; ADP, fAHP-ADP; m mAHP. Scatterplots include mean values ± SD.

For this study, human neocortical tissue samples were surgically obtained from a total of 33 patients aged 1–21 years, in three histopathological groups: patients with congenital malformations of cortical development (MCD, *n* = 13), patients with other epilepsies (OE, *n* = 15), and patients with deeply located tumors and no history of seizures (control, *n* = 5). Final pathologic diagnoses were based upon examination of epileptic tissue by a board-certified pediatric neuropathologist. Classification of focal cortical dysplasias (FCDs) was based on the ILAE classification system (Blumcke et al., 2011). The experimental tissue used in this study and the tissue sent to pathology were sent from immediately adjacent areas of lesional tissue, as determined by the attending neurosurgeon. Demographic and histopathological details are presented in Table 1. We analyzed the intrinsic properties of 108 L2/3 PNs (MCD *n* = 37; other epilepsies *n* = 45; control *n* = 26). For the initial analysis of firing patterns, intrinsic properties and AHPs, presented in Figures 1–3, L2/3 PNs from both MCD and OE subgroups are combined.

**Table 1.** Patient information

Final pathological diagnosis	Experimental group	Region of tissue resection	Patient age at surgery (years)	Patient sex
Anaplastic Pleomorphic Xanthoastrocytoma	Control	Parietal lobe	21	M
Germinoma	Control	Parietal lobe	11	M
Embryoma	Control	Temporal lobe	9	M
Atypical meningioma	Control	Parietal lobe	8	F
Glioma	Control	Temporal lobe	1	M
FCD 1B	MCD	Temporal tip	9	F
FCD IA	MCD	Lateral temporal lobe	17	F
FCD IC	MCD	Frontal lobe, temporal tip	7	M
FCD IIA	MCD	Frontal lobe	2	F
Mild MCD	MCD	Frontal lobe	3	F
Tuberous Sclerosis	MCD	Frontal lobe	10	M
FCD IIA	MCD	Occipital lobe	19	M
FCD IIB	MCD	Frontal lobe	2	F
FCD IIID	MCD	Lateral temporal lobe	6	M
FCD IIID	MCD	Temporal lobe, frontal lobe	18	M
Tuberous Sclerosis	MCD	Temporal lobe	15	M
FCD IIA	MCD	Frontal lobe	2	F
FCD IIB	MCD	Frontal lobe	7	F
Chaslin's subpial gliosis	Other epilepsy	Lateral temporal lobe	18	M
Chaslin's subpial gliosis	Other epilepsy	Lateral temporal lobe	20	M
Encephalomalacia	Other epilepsy	Frontal lobe	18	M
Chaslin's subpial gliosis	Other epilepsy	Lateral temporal lobe	5	F
Ganglioglioma	Other epilepsy	Temporal tip	16	F
Chaslin's subpial gliosis	Other epilepsy	Frontal lobe	5	M
Chaslin's subpial gliosis	Other epilepsy	Lateral temporal lobe	10	M
Gliosis and Meningioangiomatosis	Other epilepsy	Parietal lobe	16	F
Gliosis	Other epilepsy	Frontal lobe	6	M
Gliosis	Other epilepsy	Temporal lobe	5	M
Low-grade glial neoplasm	Other epilepsy	Temporal lobe	1	F
DNET	Other epilepsy	Temporal lobe	10	F
Low-grade glioma, WHO grade I	Other epilepsy	Temporal lobe	8	M
Chaslin's subpial gliosis	Other epilepsy	Temporal lobe, frontal lobe	16	M
DNET	Other epilepsy	Frontal lobe	6	F

Demographic information, diagnosis, region of tissue resection and experimental group.

10.1523/ENEURO.0247-24.2025.f2-1 Figure 2-1 **Other intrinsic properties of putative L2/3 PN subtypes.** Other analyzed intrinsic properties were graphed to show spread and variability as follows: A) resting membrane potential (RMP, mV), B) input resistance (MΩ), C) voltage sag (%), D) membrane decay, E) AHP amplitude, F) Mean FR ± SEM vs injected current (pA), G) Max FR, H) final instantaneous frequency, and I) FR accommodation ratio ± SEM vs injected current (pA). Scatter plots include mean values ± SD. Download Figure 2-1, TIF file.

10.1523/ENEURO.0247-24.2025.f2-2 Figure 2-2 **PCA and K-means clustering of epileptic putative L2/3 PNs.** A) Scree Plot used to determine the number of clusters for K-means clustering. B) Silhouette Value for each epileptic L2/3 PN based on cluster assignment. C) PCA 3D plot with neurons clustered based on 6 intrinsic properties with cluster centroids marked by an ‘X’. Download Figure 2-2, TIF file.

10.1523/ENEURO.0247-24.2025.t2-1 Table 2-1 **Post hoc test *p*-values for statistically significant properties based on putative L2/3 PN subtype.** The correlated post-hoc test for comparing intrinsic properties from Table 2. Download Table 2-1, DOCX file.

10.1523/ENEURO.0247-24.2025.t2-2 Table 2-2 **Definitions of electrophysiologic parameters.** How each intrinsic property was calculated based on the square 600ms current steps. Download Table 2-2, DOC file.

10.1523/ENEURO.0247-24.2025.f3-1 Figure 3-1 **Other intrinsic properties of L2/3 PN subtypes based on AHP shape.** Other analyzed intrinsic properties were graphed to show spread and variability as follows: A) AP half-width, B) input resistance, C) initial instantaneous frequency, D) max instantaneous frequency, E) final instantaneous frequency, and F) FR accommodation ratio. Scatter plots include mean values ± SD. Download Figure 3-1, TIF file.

10.1523/ENEURO.0247-24.2025.t3-1 Table 3-1 **Group comparison values with post hoc adjusted *p*-values for statistically significant properties based on AHP subtype.** The correlated post-hoc test for comparing intrinsic properties from Table 3. Download Table 3-1, DOCX file.

### Physiological diversity of L2/3 human epileptic neocortical PNs

In rodent neocortex, L2/3 PNs are a homogenous group of cells that exhibit a characteristic regular spiking phenotype. In contrast, human L2/3 PNs display diversity in their passive and active intrinsic properties, firing patterns, dendritic morphologies, and transcriptomes (DeFelipe and Farinas, 1992; Deitcher et al., 2017; Kalmbach et al., 2018; Berg et al., 2021). However, the diversity of human L2/3 PN physiological properties has not been examined in the setting of pediatric epilepsy. Therefore, we performed whole-cell patch-clamp electrophysiology on putative L2/3 PNs located within the epileptic focus from resected brain tissue of pediatric patients. We observed a considerable diversity in the firing patterns and action potential (AP) kinetics of these L2/3 PNs (Fig. 1). To confirm that we were successfully targeting L2/3 PNs in our epileptic tissue slices, 3D reconstructions of biocytin-filled neurons were performed on a subset of neurons (*n* = 20 reconstructions from 40 attempted fills). Although there was some variability in the extent of the dendritic arborization among the filled neurons, all L2/3 PNs filled with biocytin exhibited dendritic spines (data not shown) and morphologies consistent with PNs (Fig. 1; Mohan et al., 2015; Deitcher et al., 2017).

To determine if there were subtypes of epileptic putative L2/3 PNs, we first grouped them based on their action potential (AP) shape, afterhyperpolarization kinetics, and firing patterns at rheobase and 2× rheobase. Based on this initial categorization, we observed five distinct subtypes: accommodating neurons, regular spiking (RS) neurons, stuttering neurons, early spiking (ES) neurons, and notch neurons (Fig. 1). Accommodating neurons display significant adaptation of AP frequency and AP amplitude (Fig. 1*A*). RS neurons demonstrate the canonical RS AP shape and lack of accommodation in their firing pattern (Fig. 1*B*; Markram et al., 2015; Neske et al., 2015; Varga et al., 2015; He et al., 2016; Stedehouder et al., 2019; Berg et al., 2021; Moradi Chameh et al., 2021). Notch neurons have a fast AHP followed by an afterdepolarization (ADP; Fig. 1*C*; Foehring et al., 1991; Lorenzon and Foehring, 1992; Varga et al., 2015; Eyal et al., 2018). Stuttering neurons exhibit irregular firing patterns more commonly exhibited by interneurons (Fig. 1*D*; Druckmann et al., 2013; Stiefel et al., 2013; Komendantov et al., 2019). ES neurons exhibit a relatively hyperpolarized rheobase (Fig. 1*E*). Of the 82 recorded epileptic L2/3 PNs, we observed 7 accommodating neurons, 44 RS neurons, 18 notch neurons, 6 stuttering neurons, and 7 ES neurons (Fig. 2*A*).

After our initial classification, we analyzed the active and passive membrane properties of epileptic L2/3 PNs using a series of hyperpolarizing and depolarizing current steps (summary data and statistical analyses are presented in Table 2 and post hoc test *p* values in Extended Data Table 2-1). We observed no significant differences between PN subtypes in resting membrane potential (RMP, mV), input resistance (MΩ), or voltage sag (%) and found a significant difference in membrane decay time (ms) between accommodating and notch neurons (Extended Data Fig. 2-1*A–D*). We also observed substantial differences in AP kinetics between PN subtypes (Fig. 2*B–D*). More specifically, notch neurons showed the shortest AP half-width (ms) with a significant difference compared with accommodating neurons (Fig. 2*E*), and stuttering neurons showed the lowest spike amplitude (mV), with significant differences compared with accommodating, notch and ES neurons (Fig. 2*F*). Notch neurons displayed the shortest AHP latencies, with significant differences compared with RS and ES neurons, with RS neurons having a wide range of AHP latencies (Fig. 2*G*). We observed no subtype differences in AHP magnitude (Extended Data Fig. 2-1*E*). Analysis of the firing pattern within a train of APs revealed quantifiable differences between subtypes, as expected. In general, compared with other subtypes, we found that stuttering neurons were more likely to fire quickly initially and then accommodated their firing rates while ES neurons did not initially fire quickly but were the least likely to accommodate their firing rates (Fig. 2*H–J*). Surprisingly, we did not find differences in final instantaneous frequency (Fig. 2*K*, Extended Data Fig. 2-1*H*) or maximum firing rate (Extended Data Fig. 2-1*G*).

**Table 2.** Intrinsic properties of putative L2/3 PN subtypes based on firing properties and AP shape

Intrinsic property	Accommodating (*n* = 7, 4)	Regular Spiker (*n* = 44, 21)	Notch (*n* = 18, 10)	Stutter (*n* = 6, 4)	Early Spiker (*n* = 7, 6)	KW Test (*p* value, *H*)
Resting membrane potential (mV)	−68.47 ± 7.50	−66.53 ± 7.46	−68.33 ± 2.95	−72.86 ± 5.25	−65.77 ± 5.72	*p* = 0.1943, *H*_(4)_ = 6.07
Input resistance (MΩ)	137.60 ± 68.50	136.8 ± 74.64	88.42 ± 33.15	136.10 ± 69.14	95.73 ± 44.51	*p* = 0.1202, *H*_(4)_ = 7.31
Voltage sag (%)	5.69 ± 3.27	5.27 ± 2.59	5.22 ± 2.73	4.74 ± 2.79	4.10 ± 1.14	*p* = 0.8742, *H*_(4)_ = 1.22
Membrane decay (ms)	35.34 ± 6.99	26.49 ± 7.45	24.14 ± 5.97	24.68 ± 6.95	27.03 ± 5.94	*p* = 0.0340, *H*_(4)_ = 10.41
AP threshold (mV)	−37.57 ± 3.76	−36.31 ± 5.47	−36.84 ± 3.59	−36.87 ± 10.16	−41.50 ± 5.47	*p* = 0.2593, *H*_(4)_ = 5.28
AP amplitude (mV)	86.50 ± 7.92	79.47 ± 8.54	82.98 ± 6.30	68.67 ± 10.09	85.49 ± 6.76	*p* = 0.0016, *H*_(4)_ = 17.41
AP half-width (ms)	1.69 ± 0.43	1.30 ± 0.30	1.12 ± 0.22	1.22 ± 0.40	1.5 ± 0.34	*p* = 0.0062, *H*_(4)_ = 14.38
AHP magnitude (mV)	−15.90 ± 2.71	−14.75 ± 3.67	−15.27 ± 2.55	−14.54 ± 3.14	−16.48 ± 2.61	*p* = 0.7774, *H*_(4)_ = 1.77
AHP latency (ms)	11.56 ± 8.36	14.96 ± 12.72	3.57 ± 1.12	5.05 ± 2.14	9.10 ± 5.45	*p* < 0.0001, *H*_(4)_ = 25.45
ΔAHP (mV)	−5.12 ± 3.95	−3.72 ± 3.07	−1.89 ± 1.53	−5.53 ± 2.86	−4.46 ± 2.68	*p* = 0.0396, *H*_(4)_ = 10.05
AP broadening ratio	1.30 ± 0 0.08	1.27 ± 0.11	1.24 ± 0.10	1.32 ± 0.07	1.22 ± 0.07	*p* = 0.1265, *H*_(4)_ = 7.18
AP amplitude adaptation ratio	0.91 ± 0.06	0.93 ± 0.06	0.96 ± 0.04	0.91 ± 0.05	0.91 ± 0.05	*p* = 0.0794, *H*_(4)_ = 8.355
Initial instantaneous frequency (Hz)	19.88 ± 3.67	34.55 ± 14.20	34.05 ± 21.42	45.48 ± 13.20	17.27 ± 4.10	*p* = 0.0002, *H*_(4)_ = 22.11
Maximum firing rate (Hz)	24.35 ± 4.24	34.96 ± 11.76	32.94 ± 13.32	35.06 ± 8.37	23.69 ± 5.75	*p* = 0.0141, *H*_(4)_ = 12.49
Final instantaneous frequency (Hz)	8.10 ± 2.34	11.21 ± 3.37	9.59 ± 1.89	9.43 ± 7.67	8.48 ± 1.56	*p* = 0.0152, *H*_(4)_ = 12.31
Accommodation ratio	0.39 ± 0.10	0.37 ± 0.16	0.36 ± 0.12	0.20 ± 0.14	0.49 ± 0.11	*p* = 0.0202, *H*_(4)_ = 11.64

Intrinsic properties of accommodating, regular spiking, notch, stutter, and early spiking neurons.

Our manual classification scheme presented in Figures 1 and 2 demonstrates a high degree of physiological variability within L2/3 PNs. To determine whether these cell types could be separated mathematically, we performed an unbiased multivariable analysis using principal component analysis and *K*-means clustering using a custom-written MATLAB code. We included the six intrinsic properties that showed meaningful differences between the subtypes characterized above, including AHP latency, AP half-width, AP spike amplitude, initial ISI, FR accommodation ratio, and final ISI. We then performed a PCA using *z*-score normalized values for each property and found that three components were responsible for 76% of the total variation. Following PCA, we performed *K*-means clustering which indicated either two or five optimal clusters (Extended Data Fig. 2-2*A*). However, with five clusters, the majority of neurons (43/82) had silhouette values <0.5 (Extended Data Fig. 2-2*B*). These results indicate that PCA with *K*-means clustering did not quantifiably separate L2/3 PNs into distinct categories similar to our manually differentiated categories described above. In addition, no particular property dictated a principal component (Extended Data Fig. 2-2*C*). There are several possible explanations for the different results between manual and unsupervised clustering analyses. First, it is possible that we did not measure other variables which would separate the L2/3 PNs into classes presented in Figure 1, such as transcriptomic differences. Additionally, it is possible that the observed differences in L2/3 epileptic PN intrinsic properties have minimal physiologic significance, in terms of cellular function.

Prior studies have reported that firing pattern, firing frequency, and frequency adaptation are very highly regulated by voltage-gated potassium and calcium channels that dictate AHPs (Lorenzon and Foehring, 1992; Perez-Garci et al., 2003; Phelan et al., 2005; Higgs and Spain, 2009; Rizzo et al., 2014; Mendez-Rodriguez et al., 2021). In specific relevance to the study of epilepsy, AHP duration and/or presence of an ADP has been used to classify neurons into subtypes that differ in intrinsic excitability (Phelan et al., 2005; Mendez-Rodriguez et al., 2021). Fast AHPs (fAHPs) and medium AHPs (mAHPs) can be observed following a single action potential and during repetitive firing. In some L2/3 PNs, the fAHP is followed by an ADP which drives neuronal bursting and decreases the initial interspike interval (ISI), allowing for quicker and more powerful signal transduction (Foehring et al., 1991; Higgs and Spain, 2009). However, due to its slow kinetics, the slow AHP component (sAHP, >1 s) is only observed following a period of repetitive firing, such as a burst of APs (Rizzo et al., 2014). Thus, we did not analyze the sAHP given that the differences seen in our manual analysis were seen within the train of APs generated by a depolarization.

Given that our PCA and *K*-means clustering did not separate neurons into distinct groups based on a set of six properties, we examined whether AHP or ADP kinetics contributed to the generation of specific L2/3 PN subtypes. For this analysis, we separated neurons into three groups (Fig. 3*A*): (1) neurons with a fAHP followed by an mAHP [fAHP: 16 neurons (20.25%)], (2) neurons with a fAHP- ADP-mAHP pattern [fAHP-ADP: 28 neurons (35.44%)], and (3) neurons with only an mAHP (mAHP: 35 neurons, 44.30%). We then compared the intrinsic properties of these three subsets of neurons (mean values and statistics presented in Table 3 and post hoc test *p* values in Extended Data Table 3-1). AP kinetics indicated no significant difference in the depolarization slope (dV/dT), but we observed a significantly higher repolarization slope in fAHP neurons compared with mAHP neurons suggesting alterations in voltage-gated potassium channel conductances (Fig. 3*D*,*E*). We also observed no significant difference in AP half-widths (Extended Data Fig. 3-1*A*). As expected, mAHP neurons displayed the longest AHP latencies and also displayed the largest ΔAHP magnitudes compared with fAHP and fAHP-ADP neurons (Fig. 3*F*,*G*). These results suggest that the fAHP group of neurons may have more functional BK channels while neurons without fAHPs have less BK channels and more functional SK channels, which are known to dictate mAHP amplitude (Kohling and Wolfart, 2016).

**Table 3.** Intrinsic properties of epileptic L2/3 PNs separated based on AHP shape and latency

Intrinsic property	fAHP neurons (*n* = 16, 12)	fAHP + ADP neurons (*n* = 35, 18)	mAHP neurons (*n* = 28, 13)	Statistical comparisons (*p* value)
Resting membrane potential (mV)	−70.29 ± 5.23	−68.09 ± 7.25	−65.73 ± 6.49	KW test *p* = 0.0577
Input resistance (MΩ)	135.10 ± 53.61	104.40 ± 45.38	137.90 ± 82.44	KW test *p* = 0.2531
Voltage sag (%)	4.19 ± 2.97	5.11 ± 2.61	6.51 ± 2.63	KW test *p* = 0.0194
Membrane decay (ms)	26.89 ± 6.02	26.16 ± 7.25	27.73 ± 7.29	ANOVA *p* = 0.7114
AP threshold (mV)	−35.17 ± 6.58	−37.46 ± 4.62	−37.76 ± 5.68	KW test *p* = 0.6493
AP amplitude (mV)	78.92 ± 10.74	85.02 ± 7.40	84.98 ± 8.19	KW test *p* = 0.2345
AP half-width (ms)	1.26 ± 0.35	1.26 ± 0.36	1.40 ± 0.31	KW test *p* = 0.1375
AHP magnitude (mV)	−16.42 ± 2.74	−15.25 ± 3.56	−15.14 ± 4.01	KW test *p* = 0.6069
AHP latency (ms)	4.35 ± 1.31	4.11 ± 1.36	20.84 ± 12.36	KW test *p* < 0.0001
ΔAHP (mV)	−2.61 ± 3.06	−2.49 ± 2.47	−5.21 ± 2.91	KW test *p* = 0.0003
AP broadening ratio	1.26 ± 0.09	1.26 ± 0 0.11	1.27 ± 0.10	KW test *p* = 0.8880
AP amplitude adaptation ratio	0.93 ± 0.06	0.95 ± 0.03	0.90 ± 0.05	KW test *p* = 0.0026
Initial instantaneous frequency (Hz)	32.49 ± 15.72	33.53 ± 18.93	32.30 ± 13.25	KW test *p* = 0.9414
Maximum firing rate (Hz)	37.21 ± 13.94	35.09 ± 13.10	33.67 ± 12.03	KW test *p* = 0.6588
Final instantaneous frequency (Hz)	11.43 ± 4.71	9.78 ± 2.019	11.13 ± 4.36	KW test *p* = 0.4298
Accommodation ratio	0.38 ± 0.15	0.38 ± 0.15	0.39 ± 0.13	ANOVA *p* = 0.9230
Max depolarization slope (dV/dT)	207.80 ± 56.22	228.80 ± 39.37	206.70 ± 49.12	ANOVA *p* = 0.1265
Max repolarization slope (dV/dT)	−50.44 ± 17.06	−54.64 ± 10.75	−44.74 ± 10.48	ANOVA *p* = 0.0072

Intrinsic properties of fAHP, fAHP-ADP, and mAHP epileptic L2/3 PNs.

Surprisingly, we found that there were no significant differences between AHP subtypes in terms of firing patterns. More specifically, there were no significant differences in initial instantaneous frequency (*p* = 0.94, KW test), final instantaneous frequency (*p* = 0.43), mean instantaneous frequency (*p* = 0.66), or FR accommodation ratio (*p* = 0.08; Fig. 3*J–M*, Extended Data Fig. 3-1*C–F*, Table 3, Extended Data Table 3-1). We also note that accommodating, RS, and ES neurons included neurons that had all three types of AHP subsets. Notch neurons were made up of 83% fAHP-ADP PNs and 17% of fAHP PNs and stuttering neurons were all in the fAHP subtype (Fig. 3*N*). Together, our findings suggest that AHP and ADP kinetics do not play a significant role in determining the firing patterns of human epileptic L2/3 PNs, and therefore, the observed diversity of action potential and spiking properties are intrinsic to human epileptic L2/3 PNs and do not suggest specific subtypes of PNs with specific roles in epileptiform activity.

### L2/3 PN intrinsic properties differ between histopathologic groups

**Figure 4.**
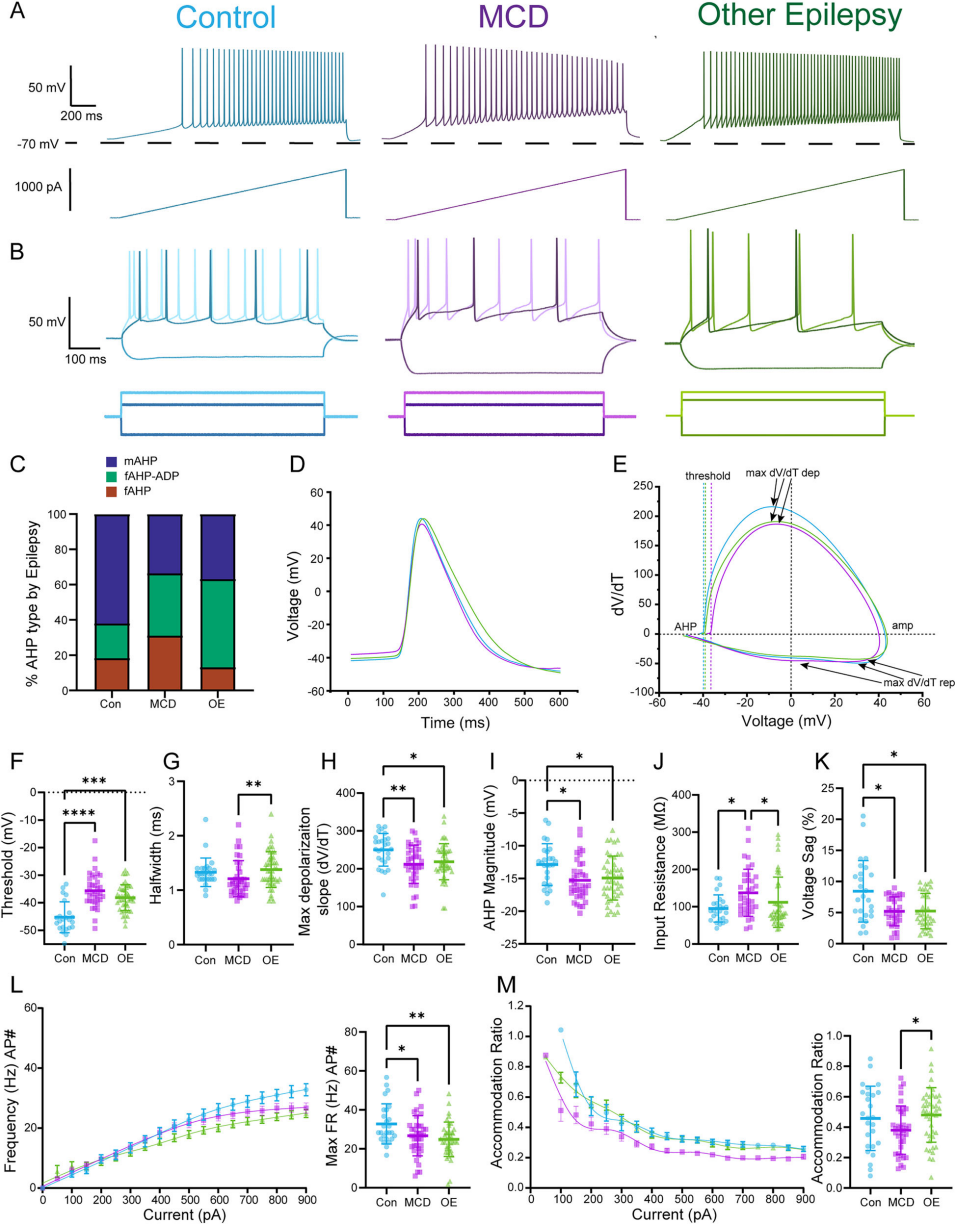
Epileptic L2/3 PNs show minor differences between etiology, but crucial differences compared with control neurons. ***A***, Representative traces from current (pA) ramp protocol (1,000 pA, 1 s) of L2/3 PNs from control (blue), MCD epileptic (purple), and other epileptic (OE) tissue (green). ***B***, Representative traces from L2/3 PNs from control and epileptic subtype showing the corresponding −250 pA hyperpolarizing step (darker negative trace), rheobase step (darker positive trace), and 2× rheobase (lighter positive trace) depolarizing current step (600 ms steps). ***C***, Percentage (%) of PNs with certain AHP cell type based on control and epilepsy subtype. ***D***, Overlay of average AP of control and epileptic subtype L2/3 PNs. ***E***, Overlay of average phase plots (dV/dT vs voltage) of control and epileptic subtype L2/3 PNs. ***F***, MCD L2/3 PNs have a depolarized AP threshold compared with control. ***G***, OE L2/3 PNs have significantly longer AP half-widths compared with MCD with trends toward longer half-widths in both epileptic subtype PNs compared with control PNs. ***H***, Depolarization slopes (dV/dT) were significantly slower in both epileptic subtypes compared with control. ***I***, AHP magnitude (mV) is significantly larger in both epileptic subtypes compared with control. ***J***, Epileptic L2/3 PNs show a wide variability in input resistance. MCD L2/3 PNs on average have significantly higher input resistances compared with control and OE L2/3 PNs. ***K***, Voltage sag is significantly smaller in both epileptic subtypes compared with control. ***L***, Left, Mean FR ± SEM versus injected current (pA). ***L***, Right, We observed lower max firing rate for both epileptic subtypes compared with control. ***M***, Left, FR accommodation ratio ± SEM versus injected current (pA). ***M***, Right, FR accommodation ratios indicate a lack of FR accommodation by OE L2/3 PNs compared with MCD L2/3 PNs with no differences compared with control. Although, on average, control PNs accommodate their frequency the most compared with the epileptic subtype PNs. Scatterplots include mean values ± SD.

In the above experiments, we grouped L2/3 PNs from patients with MCDs and other epilepsies (OEs) to demonstrate the variability of PN subtypes, given that some of the PN subtypes were relatively rare. However, we also wanted to determine if epileptic L2/3 PN intrinsic properties would differ between histopathologic groups (i.e., control, MCD, and other epilepsy). In this context, we found substantial differences in passive membrane properties and AP kinetics (mean values and statistics presented in Table 4 and post hoc test *p* values in Extended Data Table 4-1). Compared with control, we observed no significant difference in membrane decay but observed a significant decrease in voltage sag ratio (%) for both MCD and OE L2/3 PNs (Table 4, Extended Data Table 4-1, Fig. 4*K*), indicating that there may be possible alterations in *I_h_* current. Interestingly, the decrease in epileptic L2/3 PN voltage sag compared with control L2/3 PNs correlates with extensive research demonstrating altered HCN channel expression in human and rodent epilepsies (Strauss et al., 2004; Albertson et al., 2011; Brennan et al., 2016; Lin et al., 2020; Concepcion et al., 2021). In addition, we found that MCD, but not OE, L2/3 PNs had higher input resistances compared with control (Fig. 4*J*). Since we observed significant variability in epileptic L2/3 PN input resistances, and previous studies have indicated that L2/3 PN morphologies change based on distance from the pial surface, we wanted to determine if the recording distance from the pial surface could potentially be contributing to this variability (Deitcher et al., 2017). Interestingly, we observed that the input resistance did not correlate with the recorded neuron's distance from the pial surface (*r* = 0.0002, *p* = 0.8522, *n* = 18). Therefore, this variability in input resistances is most likely due to the abnormal cell types found in MCD epilepsies, such as cytomegalic/dysmorphic neurons and immature PNs (Crino, 2015; Levinson et al., 2020).

10.1523/ENEURO.0247-24.2025.t4-1 Table 4-1 **Group comparison values with post hoc adjusted *p*-values for statistically significant properties based on epilepsy subtype.** The correlated post-hoc test for comparing intrinsic properties from Table 4. Download Table 4-1, DOCX file.

**Table 4.** Intrinsic properties of L2/3 PNs split by control and epilepsy subtype

Intrinsic property	Control (*n* = 26, 5)	MCD (*n* = 37, 13)	Other epilepsies (*n* = 45, 15)	Statistical comparison (*p* value)
Resting membrane potential (mV)	−69.98 ± 1.19	−68.12 ± 7.13	−67.76 ± 5.20	ANOVA *p* = 0.3464
Input resistance (MΩ)	95.21 ± 36.54	137.20 ± 63.21	111.80 ± 67.72	KW test *p* = 0.0068
Voltage sag (%)	8.42 ± 4.95	5.20 ± 2.34	5.26 ± 2.88	KW test *p* = 0.0137
Membrane decay (ms)	26.23 ± 7.95	26.7 ± 7.37	26.77 ± 7.43	KW test *p* = 0.9238
AP threshold (mV)	−45.26 ± 5.61	−35.62 ± 6.09	−38.16 ± 4.75	KW test *p* < 0.0001
AP amplitude (mV)	84.07 ± 8.73	75.60 ± 8.37	84.64 ± 7.08	KW test *p* < 0.0001
AP half-width (ms)	1.33 ± 0.26	1.21 ± 0.33	1.38 ± 0.33	KW test *p* = 0.0064
AHP magnitude (mV)	−12.87 ± 3.19	−15.29 ± 3.19	−14.94 ± 3.36	ANOVA *p* = 0.0102
AHP latency (ms)	10.58 ± 5.12	8.92 ± 6.32	8.65 ± 5.56	KW test *p* = 0.0843
ΔAHP (mV)	−3.44 ± 3.10	−3.86 ± 3.28	−3.42 ± 2.73	KW test *p* = 0.9027
AP broadening ratio	1.23 ± 0.09	1.28 ± 0.10	1.25 ± 0.10	KW test *p* = 0.1324
AP amplitude adaptation ratio	0.95 ± 0.05	0.91 ± 0.06	0.94 ± 0.04	Welch's *p* = 0.0421
Maximum firing rate (Hz)	32.80 ± 10.29	26.68 ± 10.33	24.34 ± 8.10	ANOVA *p* = 0.0022
Accommodation ratio	0.46 ± 0.21	0.38 ± 0.16	0.48 ± 0.18	Welch's *p* = 0.0359
Maximum depolarization slope (dV/dT)	250.20 ± 44.25	211.80 ± 50.45	218.50 ± 48.12	ANOVA *p* = 0.0062
Maximum repolarization slope (dV/dT)	−53.85 ± 14.93	−52.61 ± 12.97	−48.26 ± 11.89	KW test *p* = 0.0917

Intrinsic properties of L2/3 PNs from control, MCD and OE ex vivo brain tissue.

In terms of AP kinetics and firing patterns, we found no differences in AHP latency, ΔAHP, AP amplitude adaptation, or FR accommodation between control L2/3 PNs and those from epileptic foci (Table 4, Extended Data Table 4-1). However, epileptic L2/3 PNs had the propensity to generate longer AP half-widths and had significantly depolarized thresholds, slower AP depolarization slopes, and larger AHP magnitudes (Fig. 4*F–I*). These data suggest alterations in the expression and function of perisomatic voltage-gated sodium and potassium channels including Kv1, Kv2, Kv4, SK channels, and BK channels in epileptic L2/3 PNs. Moreover, we observed a significant decrease in epileptic L2/3 PN max FRs compared with control (Fig. 4*L*, right). We postulate that although epileptic foci L2/3 PNs show signs of what is canonically considered hypoexcitable, they display trends toward longer APs and reduced activation of HCN channels. Together, this may cause heightened input summation, and therefore, more excitatory neurotransmission.

### Epilepsy etiology influences L2/3 PN properties

When comparing epileptic subtypes to one another, we noted few differences in intrinsic properties. Namely, the OE L2/3 PNs had lower input resistances, longer AP half-widths, larger AP amplitudes, and a lack of FR accommodation when compared with MCD L2/3 PNs (Table 4, Extended Data Table 4-1, Fig. 4*G*,*J*,*M*). This indicates that OE L2/3 PNs fire just as frequently (still less than control PNs) yet sustain firing with longer AP half-widths and larger AP amplitudes compared with MCD L2/3 PNs. When comparing the maximum depolarization and repolarization slopes of the AP (dV/dT), we found no significant difference between epileptic subtypes. We did find that OE L2/3 PNs had slightly faster depolarizations and slower repolarizations compared with MCD L2/3 PNs. Therefore, we postulate that these intrinsic differences may be due to dysregulated transcription, post-translational modifications, or potentially somatic mutations leading to altered function of the voltage-gated channels that regulate AP kinetics (Guerrini et al., 2015; Brennan et al., 2016; Iffland and Crino, 2017; Subramanian et al., 2019).

### 4-AP influences L2/3 PN intrinsic properties

Acute brain slices resected from epileptic foci do not generate spontaneous seizure-like activity ex vivo. Therefore, prior studies have relied on the addition of convulsant drugs to induce epileptiform activity. 4-aminopyridine (4-AP) has been used for decades as one of these convulsant drugs and works by blocking A-type potassium currents arising from the Kv1, Kv3, and Kv4 families of potassium channels (Kohling and Avoli, 2006; Guerrini et al., 2015; Williams and Hablitz, 2015; Cepeda et al., 2018; Chang et al., 2019; Levinson et al., 2020). 4-AP has been shown to block fast AHPs, increase AP half-widths, and modulate neuron firing rates and FR adaptation (Lorenzon and Foehring, 1992; Faber and Sah, 2002). However, to our knowledge, few prior studies have investigated the direct effect of 4-AP on the intrinsic properties of L2/3 PNs from epileptic human tissue. Since 4-AP is so widely used in ex vivo epilepsy electrophysiology recordings, we believed it was critical to determine whether 4-AP affects epileptic PNs differently than nonepileptic PNs, in order to adequately interpret studies (ours and others’) utilizing 4-AP to induce ictal activity in human epileptic tissue.

**Figure 5.**
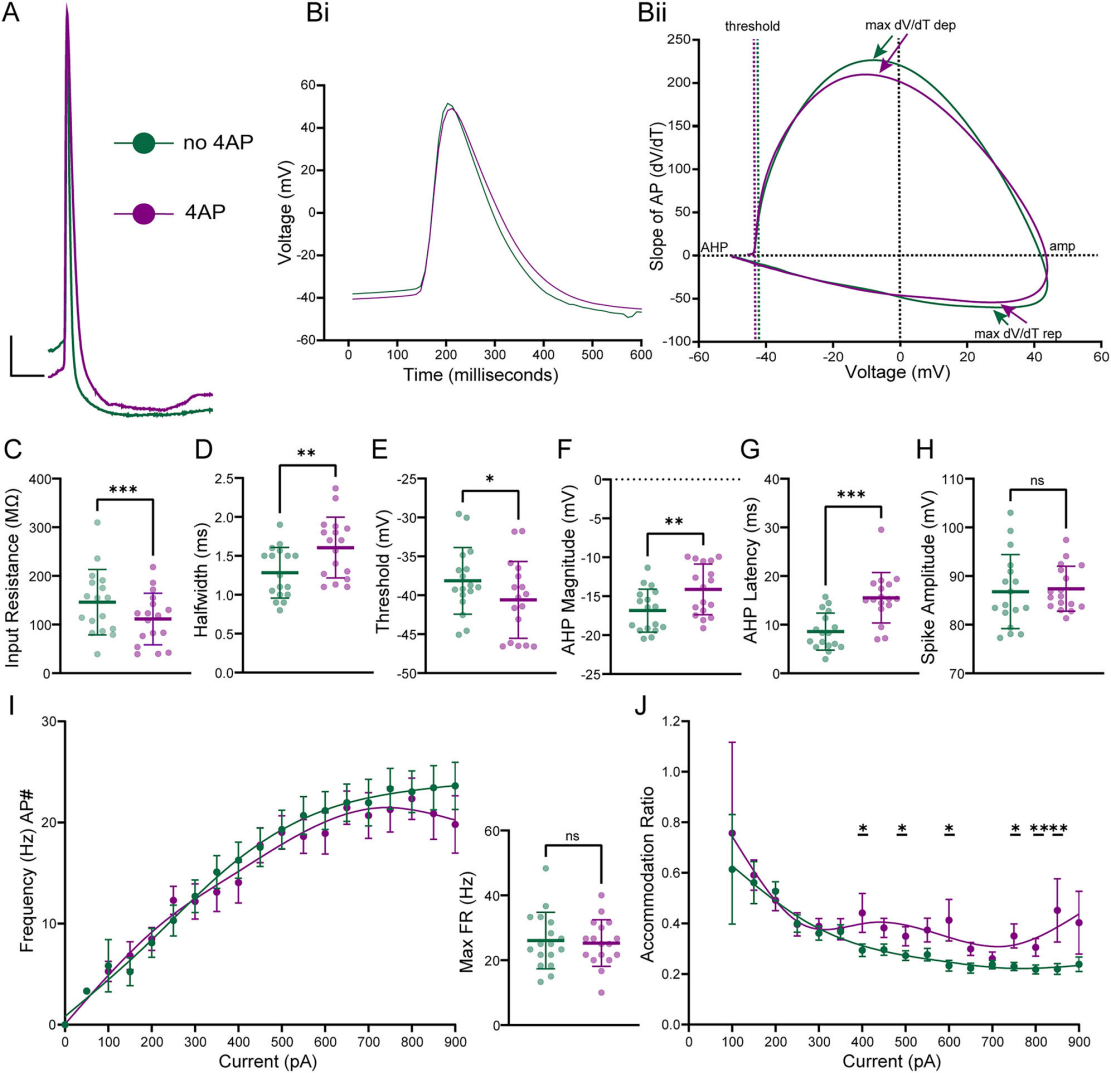
4-AP increases L2/3 PN AP half-width and AHP latency and decreases AHP magnitude leading to sustained firing. ***A***, Overlay of a representative AP before and after 4-AP wash on (scale bars: 10 mV, 10 ms). ***Bi***, Overlay of average AP before and after 4-AP wash on. ***Bii***, Overlay of phase plots. ***C***, Input resistance (MΩ) decreased after 4-AP wash on. ***D***, AP half-width increased after 4-AP wash on. ***E***, AP threshold became hyperpolarized after 4-AP wash on. ***F***, AHP magnitude was reduced after 4-AP wash on ***G***, AHP latency got slower after 4-AP wash on. ***H***, AP amplitude was not different after 4-AP wash on. ***I***, Left, Mean FR ± SEM versus injected current (pA). ***I***, Right, No significant difference was found for mean Max FR after 4-AP wash on. ***J***, FR accommodation ratio ± SEM versus injected current (pA) before and after 4-AP wash on at 400, 500, 600, 750, 800, and 850 pA depolarizing current steps indicates a significant absence of accommodation after 4-AP wash on. Scatterplots include mean values ± SD.

To this end, we performed whole-cell current-clamp recordings on L2/3 PNs and elicited hyperpolarizing and depolarizing current steps before and after 4-AP (100 μM) wash on. When comparing L2/3 PNs before and after 4-AP wash on (*n* = 17, 8), we found that 4-AP induced several alterations in epileptic L2/3 PN passive membrane properties, AP properties, and firing properties (Fig. 5, Table 5). As expected, 4-AP induced an increase in AP half-width, an increase in AHP latency, a decrease in AHP magnitude, and a hyperpolarization of the AP threshold, most likely through blockade of Kv1 and Kv4 channels located at the perisomatic region and axon initial segment (Guan et al., 2006, 2007, 2013, 2015; Pathak et al., 2016; Nguyen and Anderson, 2018; Gonzalez Sabater et al., 2021; Fig. 5*E–G*). 4-AP had no significant effect on voltage sag (%; Table 5), AP amplitude (Fig. 5*H*), or max FR (Fig. 5*I*, right). However, 4-AP did cause a subjective increase in EPSP frequency (data not shown), induced a significant decrease in L2/3 PN input resistance, and caused significant fluctuation of FR accommodation at more depolarizing potentials (400, 500, 600, 750, 800, 850 pA steps) due to ∼1-s-long sporadic hyperpolarizations (Fig. 5*C*,*J*). Lastly, we found no significant difference in the response to 4-AP between epileptic subtype L2/3 PNs [MCD (*n* = 6) and OE (*n* = 11), data not shown] suggesting that while both MCD and OE L2/3 PNs have dysfunctional Kv channel function compared with control (Fig. 4), they share similar A-Type Kv channel function (Fig. 5).

**Table 5.** 4-AP induces changes of epileptic L2/3 PN intrinsic properties

Intrinsic property	No 4-AP (*n* = 17, 8)	+4-AP (100 μM) (*n* = 17, 8)	Statistical comparisons (*p* value)
Input resistance (MΩ)	146.00 ± 67.25	111.40 ± 53.23	Paired *t* test *p* = 0.0002
Voltage sag (%)	6.43 ± 3.58	6.68 ± 3.48	Wilcoxon test *p* = 0.3060
Membrane decay (ms)	28.15 ± 7.90	33.38 ± 17.54	Wilcoxon test *p* = 0.3303
AP threshold (mV)	−38.14 ± 4.28	−40.59 ± 4.93	Paired *t* test *p* = 0.0433
AP amplitude (mV)	86.80 ± 7.62	87.40 ± 4.62	Paired *t* test *p* = 0.7729
AP half-width (ms)	1.28 ± 0.32	1.61 ± 0.39	Paired test *p* = 0.0091
AHP magnitude (mV)	−16.85 ± 2.76	−14.13 ± 3.26	Wilcoxon test *p* = 0.0021
AHP latency (ms)	8.59 ± 3.79	15.53 ± 5.19	Paired *t* test *p* = 0.0002
ΔAHP (mV)	−5.45 ± 3.22	−5.87 ± 4.50	Paired *t* test *p* = 0.6689
AP broadening ratio	1.26 ± 0.10	1.34 ± 0.25	Wilcoxon test *p* = 0.2078
AP amplitude adaptation ratio	0.91 ± 0.05	0.92 ± 0.07	Paired *t* test *p* = 0.2812
Maximum firing rate (Hz)	26.08 ± 8.70	25.29 ± 7.17	Paired *t* test *p* = 0.3543
Accommodation ratio (400 pA step)	0.29 ± 0.10	0.44 ± 0.29	Wilcoxon test *p* = 0.0273
Accommodation ratio (500 pA step)	0.27 ± 0.08	0.35 ± 0.16	Wilcoxon test *p* = 0.0342
Accommodation ratio (600 pA step)	0.23 ± 0.09	0.34 ± 0.13	Paired *t* test *p* = 0.0143
Accommodation ratio (750 pA step)	0.23 ± 0.07	0.35 ± 0.19	Wilcoxon test *p* = 0.0105
Accommodation ratio (800 pA step)	0.22 ± 0.07	0.30 ± 0.14	Wilcoxon test *p* = 0.0034
Accommodation ratio (850 pA step)	0.22 ± 0.09	0.45 ± 0.50	Wilcoxon test *p* = 0.0068

Intrinsic properties of L2/3 epileptic PNs before and after 4-AP (100 µM) wash on.

## Discussion

To our knowledge, this is the first systematic study focused on characterizing intrinsic and morphological properties of human L2/3 neocortical PNs from within the epileptic focus and comparing these to control brain tissue taken from patients without any history of epilepsy. In the present study, we demonstrate clearly distinct subtypes of L2/3 PNs in human pediatric epileptic neocortex. We demonstrate that the presence of fAHPs or mAHPs do not alone dictate the excitability or firing patterns of epileptic L2/3 PNs. Additionally, we present the new finding that human epileptic L2/3 PNs exhibit reduced voltage sag, reduced max FR, and many PNs with prolonged AP half-widths compared with control PNs. These changes in intrinsic properties suggest that L2/3 PNs may allow for more synaptic input summation and increased glutamate release from a single AP. We demonstrated that there are subtle but significant differences in the intrinsic properties of L2/3 PNS from MCD and OE groups, suggesting distinct mechanisms of epileptogenesis in MCDs. Finally, we demonstrated that 4-AP leads to an increased AP half-width, a hyperpolarized AP threshold, and increased AHP magnitude in human epileptic neurons and that epileptic subtype does not dictate L2/3 PN response to 4-AP.

### Human L2/3 PNs are intrinsically diverse

We observed that human L2/3 PNs from within the epileptic focus show a large variety of firing patterns and AP kinetics, as previously described in control L2/3 human cortical PNs (Moradi Chameh et al., 2021). Specifically, we found L2/3 PNs that exhibit regular spiking, early spiking, stuttering, and accommodating firing patterns. The last group which we termed “notch” neurons are of particular interest as they exhibit firing patterns similar to low-threshold spiking (LTS) interneurons, and yet they appear to be a subclass of PNs. Additionally, we have not encountered this cell type in mouse neocortex. In mouse cortex, LTS interneurons possess a fAHP followed by an ADP and usually express cholecystokinin (CCK), calretinin (CR), or somatostatin (SST; Beierlein et al., 2003; Tremblay et al., 2016). However, we demonstrate that morphologically, these L2/3 notch neurons are PNs. Thus, notch neurons may be a class of human L2/3 PNs not observed in mouse cortex. We postulate that the notch neuron may play a specific role in L2/3 as a network activator with the ability to vigorously respond and then adapt to a similar firing rate as RS neurons. Future studies investigating the transcriptomic profiles of the epileptic L2/3 PN subtypes, that we reported here, would help elucidate if the differences in intrinsic properties relate to gene expression profiles. Transcriptomic analysis may also provide insights into different proportions of the L2/3 PN subtypes in various subtypes of epilepsy (Berg et al., 2021).

### AHP kinetics alone do not dictate firing properties of human epileptic L2/3 PNs

AHP latency and shape has previously been thought to play a critical role in dictating firing rate and firing patterns, in association with the expression of neuronal potassium channels (Avoli and Olivier, 1989; Foehring et al., 1991; Lorenzon and Foehring, 1992; Faber and Sah, 2002; Higgs and Spain, 2009; Rizzo et al., 2014; Mendez-Rodriguez et al., 2021). Similar to previous reports on human PNs, we observed epileptic L2/3 PNs with several AHP shapes and some neurons with ADPs (Foehring et al., 1991). The differences in AHP imply differences in voltage-gated potassium channels (Kv1, Kv2, and Kv4) and/or calcium-dependent potassium channels (BK and SK channels). However, contrary to previous findings, we observed no differences in initial, maximum, or final instantaneous firing frequencies between AHP subtypes (Dodson et al., 2002; Toledo-Rodriguez et al., 2004; Guan et al., 2007, 2013; Shruti et al., 2008; Pathak et al., 2016; Rathour et al., 2016). Furthermore, we observed no differences in AHP magnitude, input resistance, or AP half-width. Our data suggest that the potassium channels that play a role in AHP shape and the corresponding firing pattern are compensatory in epileptic L2/3 PNs.

### Epilepsy etiology influences L2/3 PN properties

In this study, we included specimens from patients with TSC, HME, and FCD in the MCD epilepsies group. These epileptic subtypes are characterized by disruption of the mTOR signaling pathway leading to cortical dyslamination, neuronal heterotopia, and dysmorphic neurons (Cepeda et al., 2010, 2012; Crino, 2015). Thus, we included these pathologies within a single group to determine if PI3K-AKT-mTOR pathway mutations lead to significant changes in excitatory neuron intrinsic properties (Crino, 2011; Lasarge and Danzer, 2014; Lipton and Sahin, 2014; Blumcke and Sarnat, 2016; Hanai et al., 2017; Nguyen and Anderson, 2018; Park et al., 2018). The OEs in this study included gliosis and tumor-related epilepsies. These epilepsies are not specifically related to enhanced mTOR signaling but instead, encompass a wide range of idiopathic epilepsies with most having unclear origins (Losi et al., 2012). Gliosis is a common, but nonspecific pathological finding in patients with drug-resistant epilepsies and is characterized by the presence of reactive astrocytes, which are known to release trophic factors that promote axonal sprouting and synaptic formation (O'Dell et al., 2012). In addition, astrocytes play a crucial role in neurotransmitter concentrations and the clearance of the [K^+^]_o_ via inward rectifying K^+^ channels (Losi et al., 2012). Therefore, reactive astrocytes can facilitate improper synaptic transmission, shift neuronal excitability, and thereby, decrease seizure thresholds. The other, less common, OE cases were epileptic cases in which tumors caused seizures, such as glioma or dysembryoplastic neuroepithelial tumor (DNET). To our knowledge, there is no evidence that suggests that embryonic upregulation of the PI3K-AKT-mTOR pathway, which causes MCDs, is related to these cases, and in Type III FCDs, whether the congenital FCD occurs first or is a result of the neoplasia (Najm et al., 2018).

With the considerable differences in epilepsy subtypes, we determined the electrophysiological differences between their L2/3 PNs in order to further understand how epilepsy etiology can differ so significantly yet cause the same phenotype. With this in mind, we surprisingly observed only a few differences in L2/3 PN intrinsic properties between MCD and OEs. Specifically, we found OE L2/3 PNs had increased AP amplitudes, AP half-widths, lower input resistances, and less FR accommodation compared with MCD L2/3 PNs. This indicates that OE L2/3 PNs are effectively more hyperexcitable compared with MCD L2/3 PNs. We believe the differences compared with MCD L2/3 PNs may be due to maturation differences and the consequent abnormalities of morphologic and electrophysiologic properties. Nonetheless, our results suggest that epileptic L2/3 PNs across epileptic etiologies are more similar than different, and therefore, the cause of epilepsy may not outweigh the influences of seizure activity on excitatory neuron properties.

### Epileptic L2/3 PNs are significantly different from tumor control L2/3 PNs

Compared with control L2/3 PNs, we observed a significant decrease in max FRs in both epileptic subtype L2/3 PNs. This was surprising considering hyperexcitable neurons are usually thought to have higher FRs. We postulate this may be a homeostatic response due to seizure activity or may result from improper neuronal development, migration, and synaptic plasticity (Andreae and Burrone, 2014; Curatolo et al., 2018; Jarero-Basulto et al., 2018; Park et al., 2018; Buchsbaum and Cappello, 2019; Medvedeva and Pierani, 2020). Furthermore, we observed that many L2/3 PNs in both epileptic subtypes possessed markedly longer AP half-widths and heightened input resistances with a considerable overlap in FRs with control PNs up to 20 Hz. When taken together, we postulate that epileptic PNs may fire less when maximally stimulated but may still release more excitatory neurotransmitters (Yang and Wang, 2006).

We also observed a reduction of voltage sag in both epileptic subtypes compared with control leading us to suspect that both MCDs and OEs lead to dysregulation of HCN channel function. In neocortical PNs, HCN channels localize primarily at the dendrites and are responsible for the *I_h_* current, a nonspecific cation current that regulates the integration and summation of synaptic inputs (Brennan et al., 2016). *I_h_* has previously been shown to reduce temporal summation of synaptic inputs, and therefore, reduction of *I_h_* could lead to increased synaptic summation and contribute to network hyperexcitability (Magee, 1998; Kalmbach et al., 2018). Interestingly, dysregulated HCN channel expression and reduction in *I_h_* current have been demonstrated in both human epilepsies and rodent epilepsy models and this dysregulation is widely considered to be proepileptogenic (Strauss et al., 2004; Brennan et al., 2016).

When comparing intrinsic properties of L2/3 PNs from MCD and control cortical tissue, we found that the MCD L2/3 PNs demonstrate a more depolarized threshold and larger AHP magnitude. One possible explanation for these findings is that an increased extracellular potassium concentration has been shown to lead to a depolarized AP threshold and increased AHP magnitude (Powell and Brown, 2021). Hyperexcitable networks often lead to increased extracellular potassium due to increased numbers of APs during ictal or interictal activity (D'Antuono et al., 2004; Avoli et al., 2005; Powell and Brown, 2021). This may also occur in OE slices as their PNs also demonstrated an increased AHP magnitude. Another explanation for these AP changes would be changes in the functionality or expression of potassium channel subunits. AP threshold is modulated by Kv1 and Kv4, whereas AHP magnitude is regulated by BK and SK channels (Lorenzon and Foehring, 1992; Pineda et al., 1992; Faber and Sah, 2002; Fernandez de Sevilla et al., 2006; Guan et al., 2007; Pathak et al., 2016).

One limitation of these comparisons is the variability in recorded cortical regions. Our control tissue included parietal and temporal lobe L2/3 PN recordings while epileptic tissue recordings were made across the cerebral cortex. Previous research has found significant differences between neocortical regions in terms of PN cytoarchitectural structure and PN intrinsic properties (Jacobs et al., 2001; DeFelipe, 2011; Mohan et al., 2015; Berg et al., 2021; Moradi Chameh et al., 2021; Benavides-Piccione et al., 2024). In this study, we utilized control tissue from patients with no history of epilepsy or use of ASMs in order to decipher the influence of ictal activity on neuronal intrinsic properties. Even at our busy neurosurgical center which performs over 800 cases per year, these samples are extremely rare. Although there are differences in the cortical origin of the control, OE, and MCD groups, we believe that the variability among cortical regions is less than the variability between phenotypes. Further research is needed to gain higher resolution of PN intrinsic properties based on cortical location and epilepsy phenotype. This limitation highlights the need for multicenter collaboration and online sharing of data in a standardized form.

### 4-AP influences L2/3 PN active and passive membrane properties

Prior studies on the influence of 4-AP on human neocortical neurons have shown that 4-AP abolishes the fast AHP and increases AP half-width through blockade of perisomatic Kv1 and Kv4 channels. We observed a similar effect, as 4-AP caused an increase in AP half-widths, increased AHP latencies, and decreased AHP magnitudes. To our surprise, we observed a significant decrease in input resistances after 4-AP application, which is contrary to previous reports of increased input resistance following blockade of A-type potassium channels (Rathour et al., 2016). The mechanism behind the decreased input resistances following 4-AP wash on is currently unclear but may be due to the hyperpolarizing events that occurred during current step recordings. Overall, with the increase in AP half-width, and no significant change in AP amplitude, 4-AP seemingly elicits L2/3 PNs to fire longer APs at the same efficiency most likely leading to an increase in glutamatergic neurotransmission, thereby promoting network hyperexcitability (D'Antuono et al., 2004; Avoli et al., 2005; Yang and Wang, 2006; Abdijadid et al., 2015; Dossi et al., 2024).

### Potassium channels: antiseizure medications (ASMs)

Our data indicate that epileptic L2/3 PNs during pro-ictal, 4-AP administration display increased AP half-widths, hyperpolarized AP threshold, slower AHPs, and increased sustained firing at depolarized potentials. Therefore, increasing the activity of voltage-gated potassium channels at resting membrane potentials and/or stabilizing extracellular potassium could be beneficial therapeutic goals to reduce firing probability, decrease AP half-widths, and mitigate a hyperpolarized shift in AP threshold. Obviously, finding a single drug that will restore dynamic potassium channel function will be tricky. For example, there are over 80 genes for potassium channels in the human genome and previous reports indicate that both gain-of-function and loss-of-function mutations can cause seizures (Kohling and Wolfart, 2016; Zhao et al., 2024). However, as genetic testing becomes standard-of-care in patients with drug-resistant epilepsy, potassium channels should not be overlooked. Therapies aimed at correcting specific potassium channelopathies may provide significant benefit to patients.

## Synthesis

Reviewing Editor: Jean Christophe Poncer, INSERM Decisions are customarily a result of the Reviewing Editor and the peer reviewers coming together and discussing their recommendations until a consensus is reached. When revisions are invited, a fact-based synthesis statement explaining their decision and outlining what is needed to prepare a revision will be listed below. The following reviewer(s) agreed to reveal their identity: Karri Lämsä.

Both the reviewers and I found your work to be highly original and important to the field. However, a number of issues were raised that need to be addressed in a revised version of your manuscript.

In particular,

- The control dataset (n=2) is very limited in size, which compromises the power of statistical comparison with the epileptic tissues. Although it may be difficult to supplement your dataset in a reasonable timeframe, we suggest that you may compare your data with the available dataset from Berg et al Nature 2021 and discuss the potential limitations in comparing neurons from control and epileptic tissues.

In addition, both reviewers found the rationale for categorizing neurons into subgroups unclear and in need of further clarification. Similarly, the rationale for the 4AP experiment is not very clear and needs better justification.

Overall, the reviewers found that the manuscript is not very easy to read and suggest you put some efforts in clarifying your main findings and put them into perspective.

## Author Response

Authors' Rebuttal for Manuscript Entitled: "Characterizing the diversity of layer 2/3 human neocortical neurons in pediatric epilepsy," Synthesis of Reviews:

Both the reviewers and I found your work to be highly original and important to the field. However, a number of issues were raised that need to be addressed in a revised version of your manuscript.

In particular, the control dataset (n=2) is very limited in size, which compromises the power of statistical comparison with the epileptic tissues. Although it may be difficult to supplement your dataset in a reasonable timeframe, we suggest that you may compare your data with the available dataset from Berg et al Nature 2021 and discuss the potential limitations in comparing neurons from control and epileptic tissues.

In addition, both reviewers found the rationale for categorizing neurons into subgroups unclear and in need of further clarification. Similarly, the rationale for the 4AP experiment is not very clear and needs better justification.

Overall, the reviewers found that the manuscript is not very easy to read and suggest you put some efforts into clarifying your main findings and put them into perspective.

Response:

Thank you for the kind words and extremely helpful and detailed feedback. We have added 16 new L2/3 PNs to our control group from 3 new cases (n = 26 total) in order to increase the power and rigidity of our study. Below, and in the manuscript, we try to make clear our rationale for only using our data for our analysis and comment on how our data compares to previous publications. We also added more details about how we classified these neurons initially and included a multivariable analysis of the epileptic L2/3 PN intrinsic properties. The influence of 4-AP on intrinsic properties was included in this study as it has been used regularly to elicit epileptiform activity without directly activating or inhibiting ionotropic or metabotropic receptors allowing us to investigate underlying neurotransmission. In addition, we have added a few more details about 4-AP throughout the manuscript and include how blockade of specific ion channels influences neuronal excitability (see related details below). Lastly, we understand well that this type of study is not easy to report on due to the number of intrinsic property differences, but believe we clarified our main findings and added how they play a role in epileptogenesis. Finally, we have significantly revised the manuscript for clarity. Below, we have included our response to each comment and give some direction to where specific updates can be found.

Reviewer 1 Comments:

The manuscript provides a comprehensive assessment of the diversity of L2 L3 pyramidal neurons in epilepsy, as well as across different epilepsy types, and investigates the effect of 4-AP. They quantify several basic electrophysiological properties of the neurons and compare them amongst the various groups. Investigating the impact of pathology and 4AP on neuronal properties is of importance, and they present interesting results. However, I do have several suggestions that I feel should be addressed:

Figure 1/2: I am wondering how the electrophysiological types were distinguished, as this is not clearly conveyed in text. Could you provide more details on the criteria used? The authors missed the opportunity to emphasize the main advance of distinguishing cell types over addressing diversity. Using a multi-variable analysis method to explore the relationship between many properties might be beneficial.

Response:

We added more detail to clarify the criteria used for our initial subtype categorization. We also added a multivariable analysis (PCA analysis followed by K-means clustering, using intrinsic properties that were significantly different between categories). This clustering analysis and the corresponding data, found in Fig. 2-2, indicated that this clustering algorithm does not accurately differentiate neurons into completely independent clusters. This finding is similar to the results of Figure 2.

The results section (page 11) now includes the following section describing the results of the PCA analysis: "Our manual classification scheme presented in Figures 1 and 2 demonstrates a high degree of physiologic variability within L2/3 PNs. To determine whether these cell types could be separated mathematically, we performed an unbiased multivariable analysis using principal component analysis (PCA) and k-means clustering . We included the six intrinsic properties that showed meaningful differences between the subtypes characterized above, including: AHP latency, AP half-width, AP spike amplitude, initial ISI, FR accommodation ratio, and Final ISI. We then performed a PCA using z-score normalized values for each property and found that three components were responsible for 76% of the total variation. Following PCA, we performed K-means clustering which indicated either 2 or 5 optimal clusters (Fig.2-2A). However, with 5 clusters, the majority of neurons (43/82) had silhouette values less than 0.5 (Fig. 2-2B). These results indicate that PCA with k-means clustering does not divide the neurons into distinct categories similar to our manually differentiated categories described above. In addition, no single property dictated a principal component (Fig. 2-2C). There are several possible explanations for the different results between manual and unsupervised clustering analyses. First, it is possible that we did not measure the variables which separate the L2/3 neurons into classes presented in Fig. 1. For example, we did not analyze transcriptomic differences among the cells. Additionally, it is possible that these differences between L2/3 epileptic neurons have minimal physiologic significance in terms of cellular function." Also, please verify the scalebar in Figure 1, the 100 microns seem on the smaller side for known layer borders in human.

Response: The 100uM scale bar was based on the x axis of the biocytin fill and not the Y axis. It has been changed in the figure to denote 200uM now, which is similar to previous publications. The location of each neuron in the designated layers was based on how far away from the pial surface they are in respect to each other and the layers were representative and not to scale. We understand this might be confusing, so we opted to take out the layer indicators in Figure 1.

Figure 3: The rationale behind dividing AHP groups into f-AHP, m-AHP, and f-AHP + ADP is unclear. Can you explain why these specific categories were chosen and why no s-AHP was found? Has the difference in instantaneous frequency (Hz) of the three groups at the highest current injections been compared? Pooling data after identifying electrophysiological subtypes with different characteristics seems counterintuitive-could you clarify this rationale? Response: AHP and ADP kinetics have been used previously to differentiate neurons into functional subtypes. In addition, fast ADP has been shown to trigger burst firing, which occurs coincidentally with epileptiform activity (Kohling and Avoli, 2006). We wanted to determine if the neurons with fast AHPs and ADPs would be more likely to be detector neurons (could quickly fire to propagate excitatory neurotransmission), and with the addition of 4-AP, could transition into burst firing. We did not analyze the sAHP because this subtype of AHPs occurs on the several-second time frame. Therefore, the sAHPs do not occur between spikes in a train of events, but only at the end, which was not part of our analysis.

We have addressed this on pages 11-12: "Prior studies have reported that firing pattern, firing frequency, and frequency adaptation are very highly regulated by voltage-gated potassium and calcium channels that dictate AHPs (Lorenzon and Foehring, 1992; Perez-Garci et al., 2003; Phelan et al., 2005; Higgs and Spain, 2009; Rizzo et al., 2014; Mendez-Rodriguez et al., 2021). In specific relevance to the study of epilepsy, AHP duration and/or presence of an afterdepolarization (ADP) can be used to classify neurons into subtypes that differ in intrinsic excitability (Phelan et al., 2005; Mendez-Rodriguez et al., 2021). Fast AHPs (fAHPs) and medium AHPs (mAHPs) can be observed following a single action potential and during repetitive firing. In some L2/3 neocortical PNs, the fAHP is followed by an ADP which drives neuronal bursting and decreases the initial interspike interval (ISI), allowing for quicker and more powerful signal transduction (Foehring et al., 1991; Higgs and Spain, 2009). However, due to its slow kinetics, the slow AHP component (sAHP, > 1 s) is only observed following a period of repetitive firing such as a burst (Rizzo et al., 2014). Thus, we did not analyze the sAHP given that the differences seen in our manual analysis were seen within the train of APs generated by a depolarization.

Given that our PCA and k-means clustering did not separate neurons into distinct groups based on a set of six firing properties, we examined whether AHP or ADP kinetics contribute to the generation of specific L2/3 PN subtypes. For this analysis, we separated neurons into three groups (Fig. 3A): 1) neurons with a fAHP followed by an mAHP (fAHP: 16 neurons (20.25%)), 2) neurons with a fAHP -> ADP -> mAHP pattern (fAHP-ADP: 28 neurons (35.44%)), 3) neurons with only an mAHP (mAHP: 35 neurons, (44.30%). We then compared the intrinsic properties of these three subsets of neurons (mean values and statistics presented in Table 3)." Figure 4: The different effects of pathology on electrophysiology are interesting. However, how can you ensure that the control (n=2, parietal cortex) accurately represents control data? Is there additional cortical data from other regions to verify this, and do you consider regional variation? Response: The scarcity of true control data in studies on human epileptic tissue always presents a conundrum in terms of experimental design. Due to obvious ethical concerns, true control tissue (i.e., age-matched, location-matched, and from patients with no neurological disorders) will never be available. By using tissue from patients without epilepsy who underwent surgery to remove deeply-seated lesions, we feel that we devised the best feasible alternative to true controls.

In this revised manuscript, we have included three new cases of control tissue and now have a total of 26 control L2/3 PNs from 5 patients included in our analyses. In three patients, the tissue was obtained from the parietal lobe and in the remaining two, the tissue was obtained from the temporal lobe. Regional variability was not included in this analysis as we would need more samples from both control, MCD, and OE groups in order to have enough statistical power to analyze this additional variable.

Of course, we would like to compare our data with other publications, however, most tissue deemed "control" tissue in previous publications is actually cortical tissue from patients with epilepsy that is adjacent to the epileptic focus, which we believe is influenced by the insult of epileptic seizures. Moreover, each prior study used different solutions, recorded at different temperatures, recorded in different layers, and analyzed their properties differently. We address this in our discussion section.

The finding of higher halfwidth in OE raises questions about whether these differences coincide with upstroke and/or downstroke differences (initial examination of F4D suggests downstroke).

Response: We found no significant difference in maximum depolarization or repolarization slopes (dV/dT) when comparing OE and MCD L2/3 neurons. However, the average action potential does look like OE L2/3 PNs have longer downstrokes as seen in Fig. 4D. We address this on pages 14-15.

On a smaller note: have you considered potential variability in depth of recorded neurons (pia soma distance) in relation to the difference found in input resistance? Clarifying this might enhance your finding that disease type affects input resistance rather than variability Response: We ran a simple linear regression to determine whether there was a correlation between pia-soma distance and input resistance and found no significant correlation. We have addressed this on the bottom of page 13. "We found significant variability in epileptic L2/3 input resistance. Previous studies have shown that L2/3 morphology changes with distance from the pial surface, which could potentially contribute to alterations in input resistance (Deitcher et al., 2017). In this study, the input resistance did not correlate with the recorded neuron's distance from the pial surface (r = 0.0002, p =0.8522, n = 18)." Minor note: The figures would benefit from consistent use of colors, opacity, symbol size and units.

Response: Thank you for the suggestion, this has been addressed for consistency throughout the manuscript.

Reviewer 2 Comments with Answers:

The study has a highly original dataset, the experiments are carefully performed and reported in every detail, and statistical analysis is solid. There are certain major issues in the manuscript, and it would be important to solve them because of the rarity of this kind of data; this research study can be a landmark for human neocortex pediatric whole-cell recordings from supragranular layer pyramidal cells if revised carefully.

Main points:

1.It is pity that the reference group has only data from two patients. I understand that this type of research work can be particularly slow, but if the reference population set had statistically more powerful dataset it would have increased value of this manuscript a lot. It allows stronger comparison of parameters between epilepsy and the control groups. In addition, larger control group data would allow comparison of PC properties between early age (non-epileptic data here) and adulthood (reference data in literature).

Response: We believe we were able to address this by adding data from an additional three control patients, including 16 new L2/3 PNs. We now have data from 5 control patients and 26 PNs. The only major change in the data with the addition of these cells is that there is no longer a significant difference in membrane decay between control and epileptic groups.

2. 4-AP part is not well linked to rest of the story. Justification for these experiments is strictly speaking weak. Please strengthen this both in the introduction and discussion. One possibility is to argue that "there are functional and active Kv1 channels in PC plasma membrane already at this age in children because 100 micromolar 4-AP lowers AP firing threshold potential significantly; The AP threshold negative shift is easiest to explain by 4-AP's inhibitory effect on Kv1.1 and Kv1.2 channels which in pyramidal cells locate at perisomatic region and in the axon initial segment, which both control the firing threshold".

Response: Thank you for giving us the opportunity to clarify our justification and rationale. Briefly, 4-AP is a commonly-used agent to induce ictal activity in ex vivo slices in control rodent tissue and epileptic human tissue. We have added some information about 4-AP and why we are interested in seeing its influence on L2/3 PN intrinsic properties both in the introduction (page 3) and in the results (pages 15-16):

Introduction: "Despite the intrinsic hyperexcitability of human brain tissue resected during epilepsy surgery, it is nevertheless necessary to induce ictal activity in slices with drugs or alterations in the concentration of extracellular cations (Chang et al., 2019). One reproducible method for the generation of ictal and inter-ictal epileptiform activity in rodent or human tissue is the addition of 4-aminopyridine (4-AP), an A-type K+ channel blocker, to the perfusate (Chesnut and Swann, 1988; Avoli and Jefferys, 2016; Chang et al., 2019). The addition of 4-AP has been shown to increase action potential (AP) duration, elicit a large increase in extracellular [K+] and intracellular [Ca2+], generate a GABAergic, long-lasting depolarization, reduce firing rate accommodation, and augment neurotransmitter release leading to interictal and ictal discharges (Lorenzon and Foehring, 1992; Kohling and Avoli, 2006; Wu et al., 2009; Abdijadid et al., 2015; Williams and Hablitz, 2015). Moreover, 4-AP allows for the examination of how voltage-gated K+ channels (members of the Kv1, Kv3, and Kv4 families) play a role in hyperexcitability as 4-AP has little to no effect on Na+ or Ca2+ channels (Wulff and Zhorov, 2008). Although 4-AP can elicit ictal activity in brain slices both from control and epileptic tissue, especially in rodent models, there has been little prior study of the differing effects of 4-AP on these sets of tissue. Therefore, we examined the effects of 4-AP on L2/3 PN intrinsic properties to gain a better understanding of how this potent convulsant works to elicit ictal activity ex vivo, specifically in human epileptic tissue." Results: "Acute brain slices resected from epileptic foci do not generate spontaneous seizure-like activity ex vivo. Thus, most prior studies have relied on the addition of convulsant drugs to the ACSF to induce epileptiform activity. 4-aminopyridine (4-AP) has been used for decades as a convulsant drugs and works by blocking A-type potassium currents arising from the Kv1, Kv3, and Kv4 families of potassium channels (Kohling and Avoli, 2006; Guerrini et al., 2015; Williams and Hablitz, 2015; Cepeda et al., 2018; Chang et al., 2019; Levinson et al., 2020). 4-AP has been shown to block fast AHPs, increase AP half-widths, and modulate neuron firing rates and FR adaptation (Lorenzon and Foehring, 1992; Faber and Sah, 2002). However, to our knowledge, few prior studies have investigated the direct effect of 4-AP on the intrinsic properties of L2/3 PNs from epileptic human tissue. Since 4-AP is so widely used in ex vivo epilepsy electrophysiology recordings, we feel that it is critical to determine whether 4-AP affects epileptic PNs differently than non-epileptic PNs, in order to adequately interpret studies (ours and others') utilizing 4-AP to induce ictal activity in human epileptic tissue." 3. How many of the visualized PCs had spines in the dendrites (other than just filopodial spine type)? Although it is irrelevant here to calculate spine density, "spiny dendrites" provide a key structural feature that characterizes PCs whereas they are absent (except filopodial ones) in interneurons in layer 2/3. To confirm their presence (or absence) would give a very useful criterion to identify pyramidal cells.

Response: The L2/3 PNs visualized in this study do have dendritic spines of varying size and complexity and these details have been added to the results section (page 9). "Although there was some variability in the extent of the dendritic arborization among the filled neurons, all L2/3 PNs filled with biocytin exhibited dendritic spines (data not shown) and morphologies consistent with pyramidal neurons (Fig. 1) (Mohan et al., 2015; Deitcher et al., 2017) ." 4. The parts in discussion which make connections between specific ion channels and certain electrophysiological differences should be emphasized. The discussion is long in its current form, and general parts can be shortened. Would the findings allow to speculate different effects of specific antiepileptic drugs reported in clinical cases of these epilepsy types? Please see new ASM section in the discussion for our take on this question. "Potassium Channels: Anti-Seizure Medications (ASMs) Our data indicate that epileptic L2/3 PNS display increased AP halfwidth, hyperpolarized AP threshold, and increasing firing at depolarized potentials. Therefore, increasing the activity of voltage-gated potassium channels at resting membrane potentials and/or stabilizing extracellular potassium could be beneficial therapeutic goals to reduce firing probability, decrease AP halfwidths, and mitigate a hyperpolarized shift in AP threshold. Obviously, finding a single drug that will restore dynamic potassium channel function will be tricky. For example, there are over 80 genes for potassium channels in the human genome and previous reports indicate that both gain-of-function and loss-of-function mutations can cause seizures (Kohling and Wolfart, 2016; Zhao et al., 2024). However, as genetic testing becomes standard-of-care in patients with drug-resistant epilepsy, potassium channels should not be overlooked. Therapies aimed to correct specific potassium channelopathies may provide significant benefit to patients." 5. Please report on page 9 in Result shortly, and with references, what way the histopathological groups were defined and established? We have our tissue examined by a board-certified neuropathologist. This is now addressed in the results section on pages 8-9. "Final pathologic diagnoses were based upon examination of epileptic tissue by a board-certified pediatric neuropathologist. Classification of focal cortical dysplasias (FCD) was based on the ILAE classification system {Blumcke et al, 2011). The experimental tissue used in this study and the tissue sent to pathology were sent from immediately adjacent areas of lesional tissue, as determined by the attending neurosurgeon. " Minor:

Check that pyramidal cell is consistently abbreviated in text throughout the manuscript.

Thank you for catching this. It has been fixed! Please tone down last sentences in introduction. Remove "comprehensive," and use "systematic", for instance. In addition reformulate ...first to fully characterize...", remove "fully".

We have tried not to overstate our research and have reworded these statements based on your feedback, please see page 4 and 16. "Here, we present here a systematic analysis of the diversity of L2/3 PNs in human epileptic tissue. This study, to the best of our knowledge, is the first to characterize the diversity of L2/3 PNs from human epileptic foci including the differences in firing rate properties, passive membrane properties and AP kinetics, to show differences between L2/3 PN intrinsic properties between MCD epilepsies and other epilepsies, to determine if L2/3 PN intrinsic properties significantly differ between MCD and other epilepsies, and to show the direct effect of 4-AP on epileptic L2/3 PN intrinsic properties." Please check for typos e.g., on page 9 there is "firing pattern" written twice.

Thank you for catching this. It has been fixed!

## References

[B1] Aaberg KM, Gunnes N, Bakken IJ, Lund Soraas C, Berntsen A, Magnus P, Lossius MI, Stoltenberg C, Chin R, Suren P (2017) Incidence and prevalence of childhood epilepsy: a nationwide cohort study. Pediatrics 139:e20163908. 10.1542/peds.2016-3908 28557750

[B2] Abdijadid S, Mathern GW, Levine MS, Cepeda C (2015) Basic mechanisms of epileptogenesis in pediatric cortical dysplasia. CNS Neurosci Ther 21:92–103. 10.1111/cns.12345 25404064 PMC4442638

[B3] Albertson AJ, Yang J, Hablitz JJ (2011) Decreased hyperpolarization-activated currents in layer 5 pyramidal neurons enhances excitability in focal cortical dysplasia. J Neurophysiol 106:2189–2200. 10.1152/jn.00164.2011 21795624 PMC3214088

[B4] Andreae LC, Burrone J (2014) The role of neuronal activity and transmitter release on synapse formation. Curr Opin Neurobiol 27:47–52. 10.1016/j.conb.2014.02.008 24632375 PMC4127784

[B5] Avoli M, Bernasconi A, Mattia D, Olivier A, Hwa GG (1999) Epileptiform discharges in the human dysplastic neocortex: in vitro physiology and pharmacology. Ann Neurol 46:816–826. 10.1002/1531-8249(199912)46:6<816::AID-ANA3>3.0.CO;2-O 10589533

[B6] Avoli M, Jefferys JG (2016) Models of drug-induced epileptiform synchronization in vitro. J Neurosci Methods 260:26–32. 10.1016/j.jneumeth.2015.10.006 26484784 PMC4878885

[B7] Avoli M, Louvel J, Pumain R, Kohling R (2005) Cellular and molecular mechanisms of epilepsy in the human brain. Prog Neurobiol 77:166–200. 10.1016/j.pneurobio.2005.09.006 16307840

[B8] Avoli M, Olivier A (1989) Electrophysiological properties and synaptic responses in the deep layers of the human epileptogenic neocortex in vitro. J Neurophysiol 61:589–606. 10.1152/jn.1989.61.3.589 2709102

[B9] Beierlein M, Gibson JR, Connors BW (2003) Two dynamically distinct inhibitory networks in layer 4 of the neocortex. J Neurophysiol 90:2987–5987. 10.1152/jn.00283.2003 12815025

[B10] Benavides-Piccione R, Blazquez-Llorca L, Kastanauskaite A, Fernaud-Espinosa I, Tapia-Gonzalez S, DeFelipe J (2024) Key morphological features of human pyramidal neurons. Cereb Cortex 34:bhae180. 10.1093/cercor/bhae180 38745556 PMC11094408

[B11] Berg J, et al. (2021) Human neocortical expansion involves glutamatergic neuron diversification. Nature 598:151–158. 10.1038/s41586-021-03813-8 34616067 PMC8494638

[B12] Blauwblomme T, Dossi E, Pellegrino C, Goubert E, Iglesias BG, Sainte-Rose C, Rouach N, Nabbout R, Huberfeld G (2019) Gamma-aminobutyric acidergic transmission underlies interictal epileptogenicity in pediatric focal cortical dysplasia. Ann Neurol 85:204–217. 10.1002/ana.25403 30597612

[B13] Blumcke I, et al. (2011) The clinicopathologic spectrum of focal cortical dysplasias: a consensus classification proposed by an ad hoc task force of the ILAE Diagnostic Methods Commission. Epilepsia 52:158–174. 10.1111/j.1528-1167.2010.02777.x 21219302 PMC3058866

[B14] Blumcke I, et al. (2017) Histopathological findings in brain tissue obtained during epilepsy surgery. N Engl J Med 377:1648–1656. 10.1056/NEJMoa1703784 29069555

[B15] Blumcke I, Sarnat HB (2016) Somatic mutations rather than viral infection classify focal cortical dysplasia type II as mTORopathy. Curr Opin Neurol 29:388–395. 10.1097/WCO.0000000000000303 26840044

[B16] Brennan GP, Baram TZ, Poolos NP (2016) Hyperpolarization-activated cyclic nucleotide-gated (HCN) channels in epilepsy. Cold Spring Harb Perspect Med 6:a022384. 10.1101/cshperspect.a022384 26931806 PMC4772079

[B17] Buchsbaum IY, Cappello S (2019) Neuronal migration in the CNS during development and disease: insights from in vivo and in vitro models. Development 146:dev163766. 10.1242/dev.163766 30626593

[B18] Calcagnotto ME, Paredes MF, Tihan T, Barbaro NM, Baraban SC (2005) Dysfunction of synaptic inhibition in epilepsy associated with focal cortical dysplasia. J Neurosci 25:9649–9657. 10.1523/JNEUROSCI.2687-05.2005 16237169 PMC6725719

[B19] Camfield P, Camfield C (2015) Incidence, prevalence and aetiology of seizures and epilepsy in children. Epileptic Disord 17:117–123. 10.1684/epd.2015.0736 25895502

[B20] Cepeda C, et al. (2012) Enhanced GABAergic network and receptor function in pediatric cortical dysplasia type IIB compared with tuberous sclerosis complex. Neurobiol Dis 45:310–321. 10.1016/j.nbd.2011.08.015 21889982 PMC3225687

[B21] Cepeda C, Andre VM, Levine MS, Salamon N, Miyata H, Vinters HV, Mathern GW (2006) Epileptogenesis in pediatric cortical dysplasia: the dysmature cerebral developmental hypothesis. Epilepsy Behav 9:219–235. 10.1016/j.yebeh.2006.05.012 16875879

[B22] Cepeda C, Andre VM, Yamazaki I, Hauptman JS, Chen JY, Vinters HV, Mathern GW, Levine MS (2010) Comparative study of cellular and synaptic abnormalities in brain tissue samples from pediatric tuberous sclerosis complex and cortical dysplasia type II. Epilepsia 51:160–165. 10.1111/j.1528-1167.2010.02633.x 20618424 PMC2909023

[B23] Cepeda C, Levinson S, Yazon VW, Barry J, Mathern GW, Fallah A, Vinters HV, Levine MS, Wu JY (2018) Cellular antiseizure mechanisms of everolimus in pediatric tuberous sclerosis complex, cortical dysplasia, and non-mTOR-mediated etiologies. Epilepsia Open 3:180–190. 10.1002/epi4.12253 30564777 PMC6293070

[B24] Cepeda C, Radisavljevic Z, Peacock W, Levine MS, Buchwald NA (1992) Differential modulation by dopamine of responses evoked by excitatory amino acids in human cortex. Synapse 11:330–341. 10.1002/syn.890110408 1354399

[B25] Chang M, Dufour S, Carlen PL, Valiante TA (2019) Generation and on-demand initiation of acute ictal activity in rodent and human tissue. J Vis Exp 143:e57952. 10.3791/57952 30735161

[B26] Chesnut TJ, Swann JW (1988) Epileptiform activity induced by 4-aminopyridine in immature hippocampus. Epilepsy Res 2:187–195. 10.1016/0920-1211(88)90056-3 2848696

[B27] Concepcion FA, Khan MN, Ju Wang JD, Wei AD, Ojemann JG, Ko AL, Shi Y, Eng JK, Ramirez JM, Poolos NP (2021) HCN channel phosphorylation sites mapped by mass spectrometry in human epilepsy patients and in an animal model of temporal lobe epilepsy. Neuroscience 460:13–30. 10.1016/j.neuroscience.2021.01.038 33571596 PMC8009864

[B28] Crino PB (2011) mTOR: a pathogenic signaling pathway in developmental brain malformations. Trends Mol Med 17:734–742. 10.1016/j.molmed.2011.07.008 21890410

[B29] Crino PB (2015) Focal cortical dysplasia. Semin Neurol 35:201–208. 10.1055/s-0035-1552617 26060899 PMC6413691

[B30] Curatolo P, Moavero R, van Scheppingen J, Aronica E (2018) mTOR dysregulation and tuberous sclerosis-related epilepsy. Expert Rev Neurother 18:185–201. 10.1080/14737175.2018.1428562 29338461

[B31] D'Antuono M, Louvel J, Kohling R, Mattia D, Bernasconi A, Olivier A, Turak B, Devaux A, Pumain R, Avoli M (2004) GABAA receptor-dependent synchronization leads to ictogenesis in the human dysplastic cortex. Brain 127:1626–1640. 10.1093/brain/awh181 15175227

[B32] DeFelipe J (2011) The evolution of the brain, the human nature of cortical circuits, and intellectual creativity. Front Neuroanat 5:29. 10.3389/fnana.2011.00029 21647212 PMC3098448

[B33] DeFelipe J, Farinas I (1992) The pyramidal neuron of the cerebral cortex: morphological and chemical characteristics of the synaptic inputs. Prog Neurobiol 39:563–607. 10.1016/0301-0082(92)90015-7 1410442

[B34] Deitcher Y, Eyal G, Kanari L, Verhoog MB, Atenekeng Kahou GA, Mansvelder HD, de Kock CPJ, Segev I (2017) Comprehensive morpho-electrotonic analysis shows 2 distinct classes of L2 and L3 pyramidal neurons in human temporal cortex. Cereb Cortex 27:5398–5414. 10.1093/cercor/bhx226 28968789 PMC5939232

[B35] D'Gama AM, et al. (2017) Somatic mutations activating the mTOR pathway in dorsal telencephalic progenitors cause a continuum of cortical dysplasias. Cell Rep 21:3754–3766. 10.1016/j.celrep.2017.11.106 29281825 PMC5752134

[B36] Dodson PD, Barker MC, Forsythe ID (2002) Two heteromeric Kv1 potassium channels differentially regulate action potential firing. J Neurosci 22:6953–6961. 10.1523/JNEUROSCI.22-16-06953.2002 12177193 PMC6757903

[B37] Dossi E, Zonca L, Pivonkova H, Milior G, Moulard J, Vargova L, Chever O, Holcman D, Rouach N (2024) Astroglial gap junctions strengthen hippocampal network activity by sustaining afterhyperpolarization via KCNQ channels. Cell Rep 43:114158. 10.1016/j.celrep.2024.114158 38722742

[B38] Druckmann S, Hill S, Schurmann F, Markram H, Segev I (2013) A hierarchical structure of cortical interneuron electrical diversity revealed by automated statistical analysis. Cereb Cortex 23:2994–3006. 10.1093/cercor/bhs290 22989582

[B39] Eyal G, Verhoog MB, Testa-Silva G, Deitcher Y, Benavides-Piccione R, DeFelipe J, de Kock CPJ, Mansvelder HD, Segev I (2018) Human cortical pyramidal neurons: from spines to spikes via models. Front Cell Neurosci 12:181. 10.3389/fncel.2018.00181 30008663 PMC6034553

[B40] Faber ES, Sah P (2002) Physiological role of calcium-activated potassium currents in the rat lateral amygdala. J Neurosci 22:1618–1628. 10.1523/JNEUROSCI.22-05-01618.2002 11880492 PMC6758860

[B41] Fernandez de Sevilla D, Garduno J, Galvan E, Buno W (2006) Calcium-activated afterhyperpolarizations regulate synchronization and timing of epileptiform bursts in hippocampal CA3 pyramidal neurons. J Neurophysiol 96:3028–3041. 10.1152/jn.00434.2006 16971683

[B42] Foehring RC, Lorenzon NM, Herron P, Wilson CJ (1991) Correlation of physiologically and morphologically identified neuronal types in human association cortex in vitro. J Neurophysiol 66:1825–1837. 10.1152/jn.1991.66.6.1825 1812219

[B43] Golyala A, Kwan P (2017) Drug development for refractory epilepsy: the past 25 years and beyond. Seizure 44:147–156. 10.1016/j.seizure.2016.11.022 28017578

[B44] Gonzalez Sabater V, Rigby M, Burrone J (2021) Voltage-gated potassium channels ensure action potential shape fidelity in distal axons. J Neurosci 41:5372–5385. 10.1523/JNEUROSCI.2765-20.2021 34001627 PMC8221596

[B45] Guan D, Armstrong WE, Foehring RC (2013) Kv2 channels regulate firing rate in pyramidal neurons from rat sensorimotor cortex. J Physiol 591:4807–4825. 10.1113/jphysiol.2013.257253 23878373 PMC3800456

[B46] Guan D, Armstrong WE, Foehring RC (2015) Electrophysiological properties of genetically identified subtypes of layer 5 neocortical pyramidal neurons: Ca(2)(+) dependence and differential modulation by norepinephrine. J Neurophysiol 113:2014–2032. 10.1152/jn.00524.2014 25568159 PMC4416592

[B47] Guan D, Lee JC, Higgs MH, Spain WJ, Foehring RC (2007) Functional roles of Kv1 channels in neocortical pyramidal neurons. J Neurophysiol 97:1931–1940. 10.1152/jn.00933.2006 17215507

[B48] Guan D, Lee JC, Tkatch T, Surmeier DJ, Armstrong WE, Foehring RC (2006) Expression and biophysical properties of Kv1 channels in supragranular neocortical pyramidal neurones. J Physiol 571:371–389. 10.1113/jphysiol.2005.097006 16373387 PMC1796796

[B49] Guerrini R, et al. (2015) Diagnostic methods and treatment options for focal cortical dysplasia. Epilepsia 56:1669–1686. 10.1111/epi.13200 26434565

[B50] Guthman EM, Garcia JD, Ma M, Chu P, Baca SM, Smith KR, Restrepo D, Huntsman MM (2020) Cell-type-specific control of basolateral amygdala neuronal circuits via entorhinal cortex-driven feedforward inhibition. Elife 9:e50601. 10.7554/eLife.50601 31916940 PMC6984813

[B51] Hanai S, et al. (2017) Pathologic active mTOR mutation in brain malformation with intractable epilepsy leads to cell-autonomous migration delay. Am J Pathol 187:1177–1185. 10.1016/j.ajpath.2017.01.015 28427592

[B52] He M, et al. (2016) Strategies and tools for combinatorial targeting of GABAergic neurons in mouse cerebral cortex. Neuron 91:1228–1243. 10.1016/j.neuron.2016.08.021 27618674 PMC5223593

[B53] Heinemann U, Staley KJ (2014) What is the clinical relevance of in vitro epileptiform activity? In: *Issues in clinical epileptology: a view from the bench*, Ed 1, (Scharfman HE, Buckmaster PS, eds), pp 25–41. Dordrecht: Springer. 10.1007/978-94-017-8914-1_2 25012364

[B54] Higgs MH, Spain WJ (2009) Conditional bursting enhances resonant firing in neocortical layer 2-3 pyramidal neurons. J Neurosci 29:1285–1299. 10.1523/JNEUROSCI.3728-08.2009 19193876 PMC6666063

[B55] Huberfeld G, Menendez de la Prida L, Pallud J, Cohen I, Le Van Quyen M, Adam C, Clemenceau S, Baulac M, Miles R (2011) Glutamatergic pre-ictal discharges emerge at the transition to seizure in human epilepsy. Nat Neurosci 14:627–634. 10.1038/nn.2790 21460834

[B56] Hwa GG, Avoli M, Oliver A, Villemure JG (1991) Bicuculline-induced epileptogenesis in the human neocortex maintained in vitro. Exp Brain Res 83:329–339. 10.1007/BF00231156 1673658

[B57] Iffland PH 2nd, Crino PB (2017) Focal cortical dysplasia: gene mutations, cell signaling, and therapeutic implications. Annu Rev Pathol 12:547–571. 10.1146/annurev-pathol-052016-100138 28135561

[B58] Jacobs J, LeVan P, Chander R, Hall J, Dubeau F, Gotman J (2008) Interictal high-frequency oscillations (80-500Hz) are an indicator of seizure onset areas independent of spikes in the human epileptic brain. Epilepsia 49:1893–1907. 10.1111/j.1528-1167.2008.01656.x 18479382 PMC3792077

[B59] Jacobs B, Schall M, Prather M, Kapler E, Driscoll L, Baca S, Jacobs J, Ford K, Wainwright M, Treml M (2001) Regional dendritic and spine variation in human cerebral cortex: a quantitative Golgi study. Cereb Cortex 11:558–571. 10.1093/cercor/11.6.558 11375917

[B60] Jarero-Basulto JJ, Gasca-Martinez Y, Rivera-Cervantes MC, Urena-Guerrero ME, Feria-Velasco AI, Beas-Zarate C (2018) Interactions between epilepsy and plasticity. Pharmaceuticals 11:17. 10.3390/ph11010017 29414852 PMC5874713

[B61] Jozwiak J, Kotulska K, Jozwiak S (2006) Similarity of balloon cells in focal cortical dysplasia to giant cells in tuberous sclerosis. Epilepsia 47:805. 10.1111/j.1528-1167.2006.00531_1.x 16650151

[B62] Kalmbach BE, et al. (2018) h-Channels contribute to divergent intrinsic membrane properties of supragranular pyramidal neurons in human versus mouse cerebral cortex. Neuron 100:1194–1208.e5. 10.1016/j.neuron.2018.10.012 30392798 PMC6447369

[B63] Kohling R, Avoli M (2006) Methodological approaches to exploring epileptic disorders in the human brain in vitro. J Neurosci Methods 155:1–19. 10.1016/j.jneumeth.2006.04.009 16753220

[B64] Kohling R, Wolfart J (2016) Potassium channels in epilepsy. Cold Spring Harb Perspect Med 6:a022871. 10.1101/cshperspect.a022871 27141079 PMC4852798

[B65] Komendantov AO, Venkadesh S, Rees CL, Wheeler DW, Hamilton DJ, Ascoli GA (2019) Quantitative firing pattern phenotyping of hippocampal neuron types. Sci Rep 9:17915. 10.1038/s41598-019-52611-w 31784578 PMC6884469

[B66] Lamberink HJ, Otte WM, Blumcke I, Braun KPJ, European Epilepsy Brain Bank Writing Group, Study Group, European Reference Network EpiCARE (2020) Seizure outcome and use of antiepileptic drugs after epilepsy surgery according to histopathological diagnosis: a retrospective multicentre cohort study. Lancet Neurol 19:748–757. 10.1016/S1474-4422(20)30220-9 32822635

[B67] Lasarge CL, Danzer SC (2014) Mechanisms regulating neuronal excitability and seizure development following mTOR pathway hyperactivation. Front Mol Neurosci 7:18. 10.3389/fnmol.2014.00018 24672426 PMC3953715

[B68] Levinson S, Tran CH, Barry J, Viker B, Levine MS, Vinters HV, Mathern GW, Cepeda C (2020) Paroxysmal discharges in tissue slices from pediatric epilepsy surgery patients: critical role of GABA(B) receptors in the generation of ictal activity. Front Cell Neurosci 14:54. 10.3389/fncel.2020.00054 32265658 PMC7099654

[B69] Lin W, et al. (2020) Downregulation of hyperpolarization-activated cyclic nucleotide-gated channels (HCN) in the hippocampus of patients with medial temporal lobe epilepsy and hippocampal sclerosis (MTLE-HS). Hippocampus 30:1112–1126. 10.1002/hipo.23219 32543742

[B70] Lipton JO, Sahin M (2014) The neurology of mTOR. Neuron 84:275–291. 10.1016/j.neuron.2014.09.034 25374355 PMC4223653

[B71] Lorenzon NM, Foehring RC (1992) Relationship between repetitive firing and afterhyperpolarizations in human neocortical neurons. J Neurophysiol 67:350–363. 10.1152/jn.1992.67.2.350 1373765

[B72] Losi G, Cammarota M, Carmignoto G (2012) The role of astroglia in the epileptic brain. Front Pharmacol 3:132. 10.3389/fphar.2012.00132 22807916 PMC3395023

[B73] Magee JC (1998) Dendritic hyperpolarization-activated currents modify the integrative properties of hippocampal CA1 pyramidal neurons. J Neurosci 18:7613–7624. 10.1523/JNEUROSCI.18-19-07613.1998 9742133 PMC6793032

[B74] Markram H, et al. (2015) Reconstruction and simulation of neocortical microcircuitry. Cell 163:456–492. 10.1016/j.cell.2015.09.029 26451489

[B75] Mattia D, Olivier A, Avoli M (1995) Seizure-like discharges recorded in human dysplastic neocortex maintained in vitro. Neurology 45:1391–1395. 10.1212/WNL.45.7.1391 7617202

[B76] Medvedeva VP, Pierani A (2020) How do electric fields coordinate neuronal migration and maturation in the developing cortex? Front Cell Dev Biol 8:580657. 10.3389/fcell.2020.580657 33102486 PMC7546860

[B77] Mendez-Rodriguez BS, Arias-Garcia MA, Tapia D, Laville A, Bargas J, Galarraga E (2021) Firing differences between adult intralaminar thalamo-striatal neurons. Neuroscience 458:153–165. 10.1016/j.neuroscience.2020.12.032 33428968

[B78] Mohan H, et al. (2015) Dendritic and axonal architecture of individual pyramidal neurons across layers of adult human neocortex. Cereb Cortex 25:4839–4853. 10.1093/cercor/bhv188 26318661 PMC4635923

[B79] Moradi Chameh H, Rich S, Wang L, Chen FD, Zhang L, Carlen PL, Tripathy SJ, Valiante TA (2021) Diversity amongst human cortical pyramidal neurons revealed via their sag currents and frequency preferences. Nat Commun 12:2497. 10.1038/s41467-021-22741-9 33941783 PMC8093195

[B80] Najm IM, Sarnat HB, Blumcke I (2018) Review: the international consensus classification of focal cortical dysplasia - a critical update 2018. Neuropathol Appl Neurobiol 44:18–31. 10.1111/nan.12462 29359399

[B81] Nakagawa JM, Donkels C, Fauser S, Schulze-Bonhage A, Prinz M, Zentner J, Haas CA (2017) Characterization of focal cortical dysplasia with balloon cells by layer-specific markers: evidence for differential vulnerability of interneurons. Epilepsia 58:635–645. 10.1111/epi.13690 28206669

[B82] Neske GT, Patrick SL, Connors BW (2015) Contributions of diverse excitatory and inhibitory neurons to recurrent network activity in cerebral cortex. J Neurosci 35:1089–1105. 10.1523/JNEUROSCI.2279-14.2015 25609625 PMC4300319

[B83] Nguyen LH, Anderson AE (2018) mTOR-dependent alterations of Kv1.1 subunit expression in the neuronal subset-specific Pten knockout mouse model of cortical dysplasia with epilepsy. Sci Rep 8:3568. 10.1038/s41598-018-21656-8 29476105 PMC5824782

[B84] O'Dell CM, Das A, Wallace Gt, Ray SK, Banik NL (2012) Understanding the basic mechanisms underlying seizures in mesial temporal lobe epilepsy and possible therapeutic targets: a review. J Neurosci Res 90:913–924. 10.1002/jnr.22829 22315182 PMC11877321

[B85] Park SM, et al. (2018) Brain somatic mutations in MTOR disrupt neuronal ciliogenesis, leading to focal cortical dyslamination. Neuron 99:83–97.e7. 10.1016/j.neuron.2018.05.039 29937275

[B86] Pathak D, Guan D, Foehring RC (2016) Roles of specific Kv channel types in repolarization of the action potential in genetically identified subclasses of pyramidal neurons in mouse neocortex. J Neurophysiol 115:2317–2329. 10.1152/jn.01028.2015 26864770 PMC4922457

[B87] Perez-Garci E, Bargas J, Galarraga E (2003) The role of Ca2+ channels in the repetitive firing of striatal projection neurons. Neuroreport 14:1253–1256. 10.1097/00001756-200307010-00013 12824770

[B88] Phelan KD, Mahler HR, Deere T, Cross CB, Good C, Garcia-Rill E (2005) Postnatal maturational properties of rat parafascicular thalamic neurons recorded in vitro. Thalamus Relat Syst 3:89–113. 10.1017/S1472928805000105 19305519 PMC2658616

[B89] Pineda JC, Galarraga E, Bargas J, Cristancho M, Aceves J (1992) Charybdotoxin and apamin sensitivity of the calcium-dependent repolarization and the afterhyperpolarization in neostriatal neurons. J Neurophysiol 68:287–294. 10.1152/jn.1992.68.1.287 1381420

[B90] Powell CL, Brown AM (2021) A classic experiment revisited: membrane permeability changes during the action potential. Adv Physiol Educ 45:178–181. 10.1152/advan.00188.2020 33661050

[B91] Quiquempoix M, Fayad SL, Boutourlinsky K, Leresche N, Lambert RC, Bessaih T (2018) Layer 2/3 pyramidal neurons control the gain of cortical output. Cell Rep 24:2799–2807.e4. 10.1016/j.celrep.2018.08.038 30208307

[B92] Rathour RK, Malik R, Narayanan R (2016) Transient potassium channels augment degeneracy in hippocampal active dendritic spectral tuning. Sci Rep 6:24678. 10.1038/srep24678 27094086 PMC4837398

[B93] Rizzo V, Richman J, Puthanveettil SV (2014) Dissecting mechanisms of brain aging by studying the intrinsic excitability of neurons. Front Aging Neurosci 6:337. 10.3389/fnagi.2014.00337 25610394 PMC4285138

[B94] Rossini L, et al. (2021) Dendritic pathology, spine loss and synaptic reorganization in human cortex from epilepsy patients. Brain 144:251–265. 10.1093/brain/awaa387 33221837

[B95] Shruti S, Clem RL, Barth AL (2008) A seizure-induced gain-of-function in BK channels is associated with elevated firing activity in neocortical pyramidal neurons. Neurobiol Dis 30:323–330. 10.1016/j.nbd.2008.02.002 18387812 PMC2665726

[B96] Stedehouder J, et al. (2019) Local axonal morphology guides the topography of interneuron myelination in mouse and human neocortex. Elife 8:e48615. 10.7554/eLife.48615 31742557 PMC6927753

[B97] Stiefel KM, Englitz B, Sejnowski TJ (2013) Origin of intrinsic irregular firing in cortical interneurons. Proc Natl Acad Sci U S A 110:7886–7891. 10.1073/pnas.1305219110 23610409 PMC3651468

[B98] Strauss U, Kole MHP, Brauer AU, Pahnke J, Bajorat R, Rolfs A, Nitsch R, Deisz RA (2004) An impaired neocortical Ih is associated with enhanced excitability and absence epilepsy. Eur J Neurosci 19:3048–3058. 10.1111/j.0953-816X.2004.03392.x 15182313

[B99] Subramanian L, Calcagnotto ME, Paredes MF (2019) Cortical malformations: lessons in human brain development. Front Cell Neurosci 13:576. 10.3389/fncel.2019.00576 32038172 PMC6993122

[B100] Swietek B, Gupta A, Proddutur A, Santhakumar V (2016) Immunostaining of biocytin-filled and processed sections for neurochemical markers. J Vis Exp 118:e54880. 10.3791/54880 28117774 PMC5264554

[B101] Talos DM, Sun H, Kosaras B, Joseph A, Folkerth RD, Poduri A, Madsen JR, Black PM, Jensen FE (2012) Altered inhibition in tuberous sclerosis and type IIb cortical dysplasia. Ann Neurol 71:539–551. 10.1002/ana.22696 22447678 PMC3334406

[B102] Toledo-Rodriguez M, Blumenfeld B, Wu C, Luo J, Attali B, Goodman P, Markram H (2004) Correlation maps allow neuronal electrical properties to be predicted from single-cell gene expression profiles in rat neocortex. Cereb Cortex 14:1310–1327. 10.1093/cercor/bhh092 15192011

[B103] Tremblay R, Lee S, Rudy B (2016) GABAergic interneurons in the neocortex: from cellular properties to circuits. Neuron 91:260–352. 10.1016/j.neuron.2016.06.033 27477017 PMC4980915

[B104] Varga C, Tamas G, Barzo P, Olah S, Somogyi P (2015) Molecular and electrophysiological characterization of GABAergic interneurons expressing the transcription factor COUP-TFII in the adult human temporal cortex. Cereb Cortex 25:4430–4449. 10.1093/cercor/bhv045 25787832 PMC4768361

[B105] Williams SB, Hablitz JJ (2015) Differential modulation of repetitive firing and synchronous network activity in neocortical interneurons by inhibition of A-type K(+) channels and Ih. Front Cell Neurosci 9:89. 10.3389/fncel.2015.00089 25852481 PMC4364302

[B106] Wu ZZ, Li DP, Chen SR, Pan HL (2009) Aminopyridines potentiate synaptic and neuromuscular transmission by targeting the voltage-activated calcium channel beta subunit. J Biol Chem 284:36453–36461. 10.1074/jbc.M109.075523 19850918 PMC2794761

[B107] Wu S, Wei T, Fan W, Wang Y, Li C, Deng J (2021) Cell cycle during neuronal migration and neocortical lamination. Int J Dev Neurosci 81:209–219. 10.1002/jdn.10091 33448039

[B108] Wuarin JP, Kim YI, Cepeda C, Tasker JG, Walsh JP, Peacock WJ, Buchwald NA, Dudek FE (1990) Synaptic transmission in human neocortex removed for treatment of intractable epilepsy in children. Ann Neurol 28:503–511. 10.1002/ana.410280406 1979219

[B109] Wulff H, Zhorov BS (2008) K+ channel modulators for the treatment of neurological disorders and autoimmune diseases. Chem Rev 108:1744–1773. 10.1021/cr078234p 18476673 PMC2714671

[B110] Yang YM, Wang LY (2006) Amplitude and kinetics of action potential-evoked Ca2+ current and its efficacy in triggering transmitter release at the developing calyx of Held synapse. J Neurosci 26:5698–5708. 10.1523/JNEUROSCI.4889-05.2006 16723526 PMC6675268

[B111] Zhao T, Wang L, Chen F (2024) Potassium channel-related epilepsy: pathogenesis and clinical features. Epilepsia Open 9:891–905. 10.1002/epi4.12934 38560778 PMC11145612

