# Supplementary figures and images for: Characterizing the Diversity of Layer 2/3 Human Neocortical Neurons in Pediatric Epilepsy
Source: eNeuro. 2025 May 2;12(5):ENEURO.0247-24.2025. doi: 10.1523/ENEURO.0247-24.2025 (PMC12061357; doi:10.1523/ENEURO.0247-24.2025)

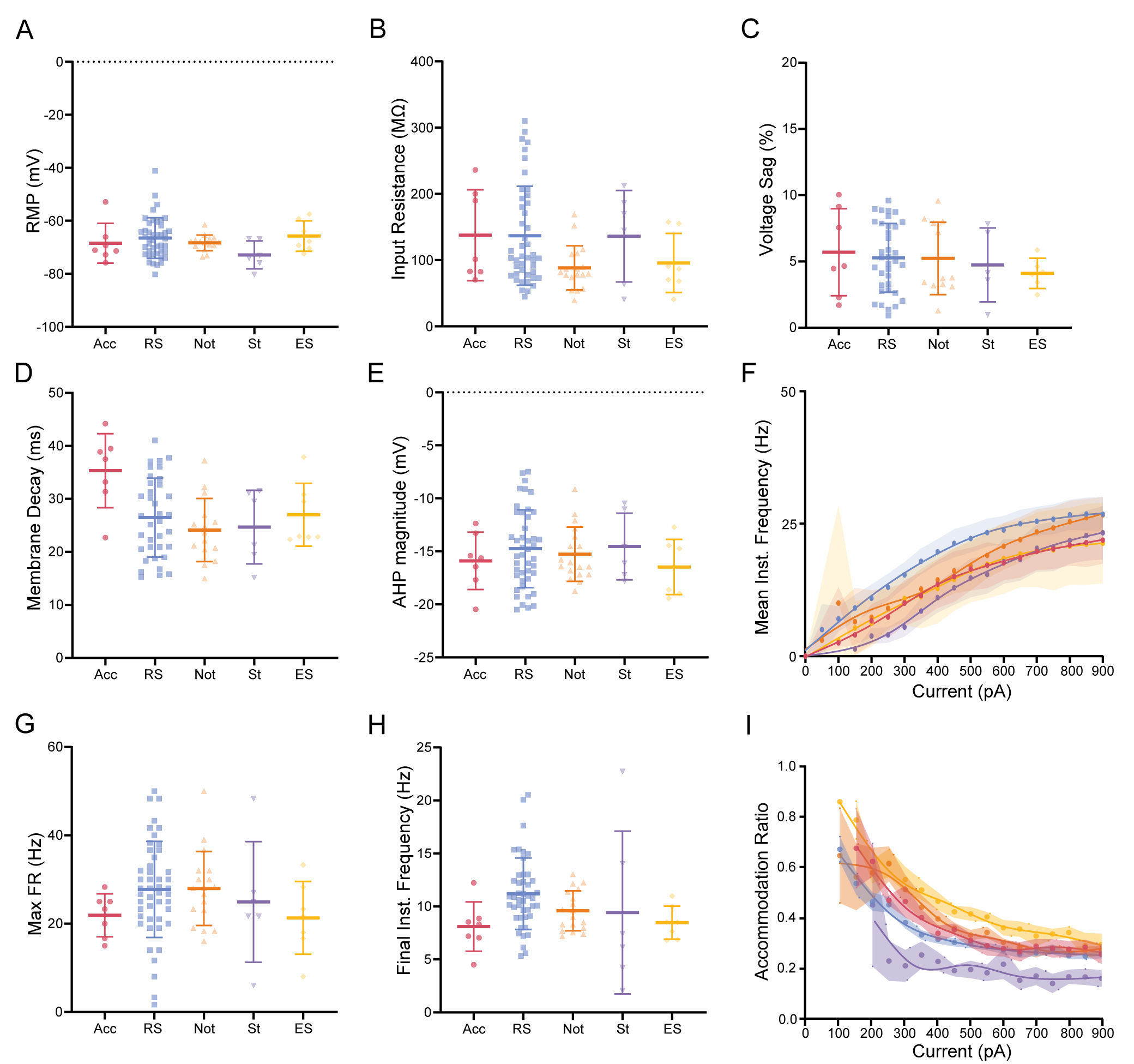

Supplement: Figure 2-1 — Other intrinsic properties of putative L2/3 PN subtypes. Other analyzed intrinsic properties were graphed to show spread and variability as follows: A) resting membrane potential (RMP, mV), B) input resistance (MΩ), C) voltage sag (%), D) membrane decay, E) AHP amplitude, F) Mean FR ± SEM vs injected current (pA), G) Max FR, H) final instantaneous frequency, and I) FR accommodation ratio ± SEM vs injected current (pA). Scatter plots include mean values ± SD. Download Figure 2-1, TIF file. [file eneuro-12-ENEURO.0247-24.2025-s001.tif]

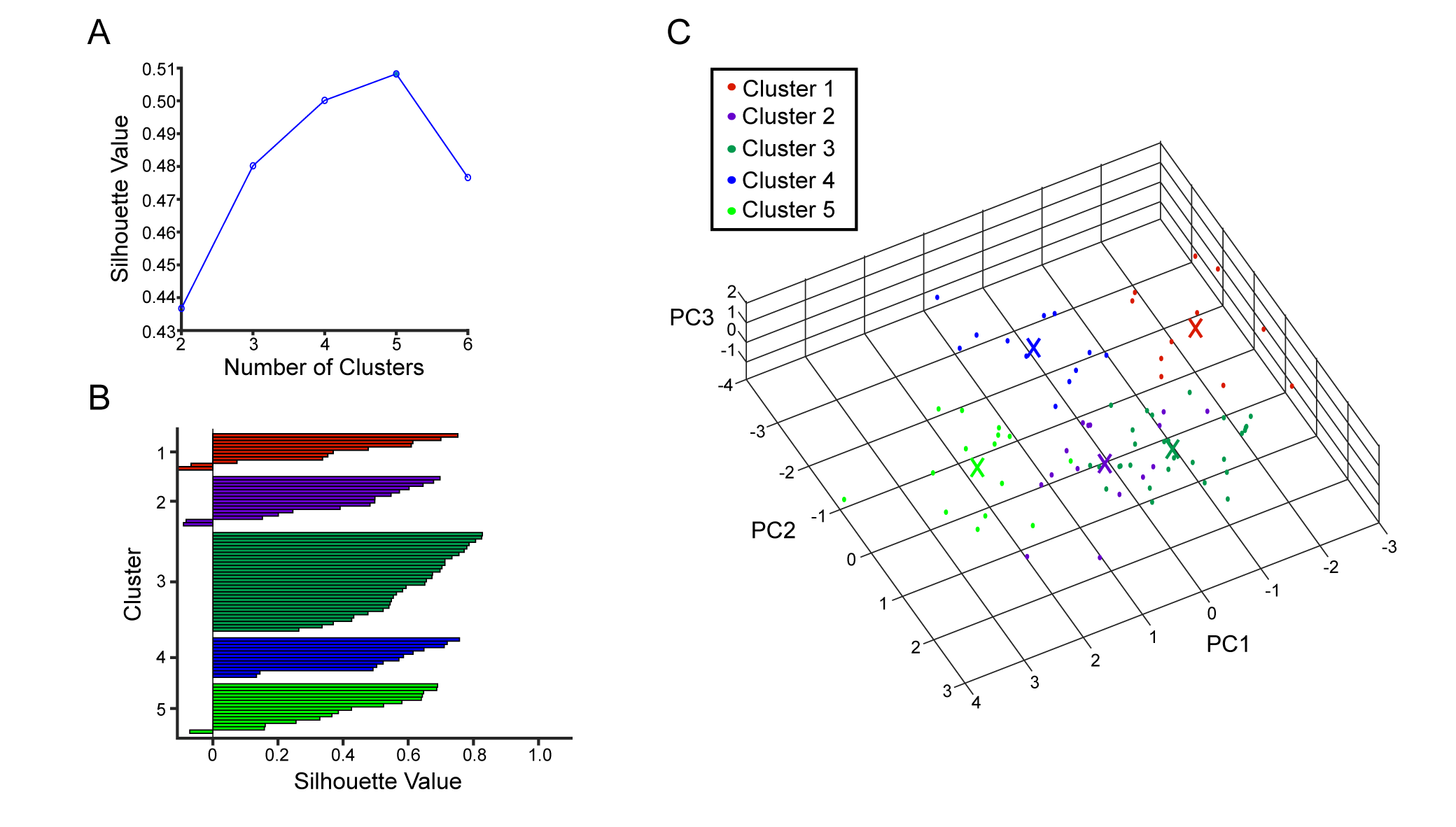

Supplement: Figure 2-2 — PCA and K-means clustering of epileptic putative L2/3 PNs. A) Scree Plot used to determine the number of clusters for K-means clustering. B) Silhouette Value for each epileptic L2/3 PN based on cluster assignment. C) PCA 3D plot with neurons clustered based on 6 intrinsic properties with cluster centroids marked by an ‘X’. Download Figure 2-2, TIF file. [file eneuro-12-ENEURO.0247-24.2025-s002.tif]

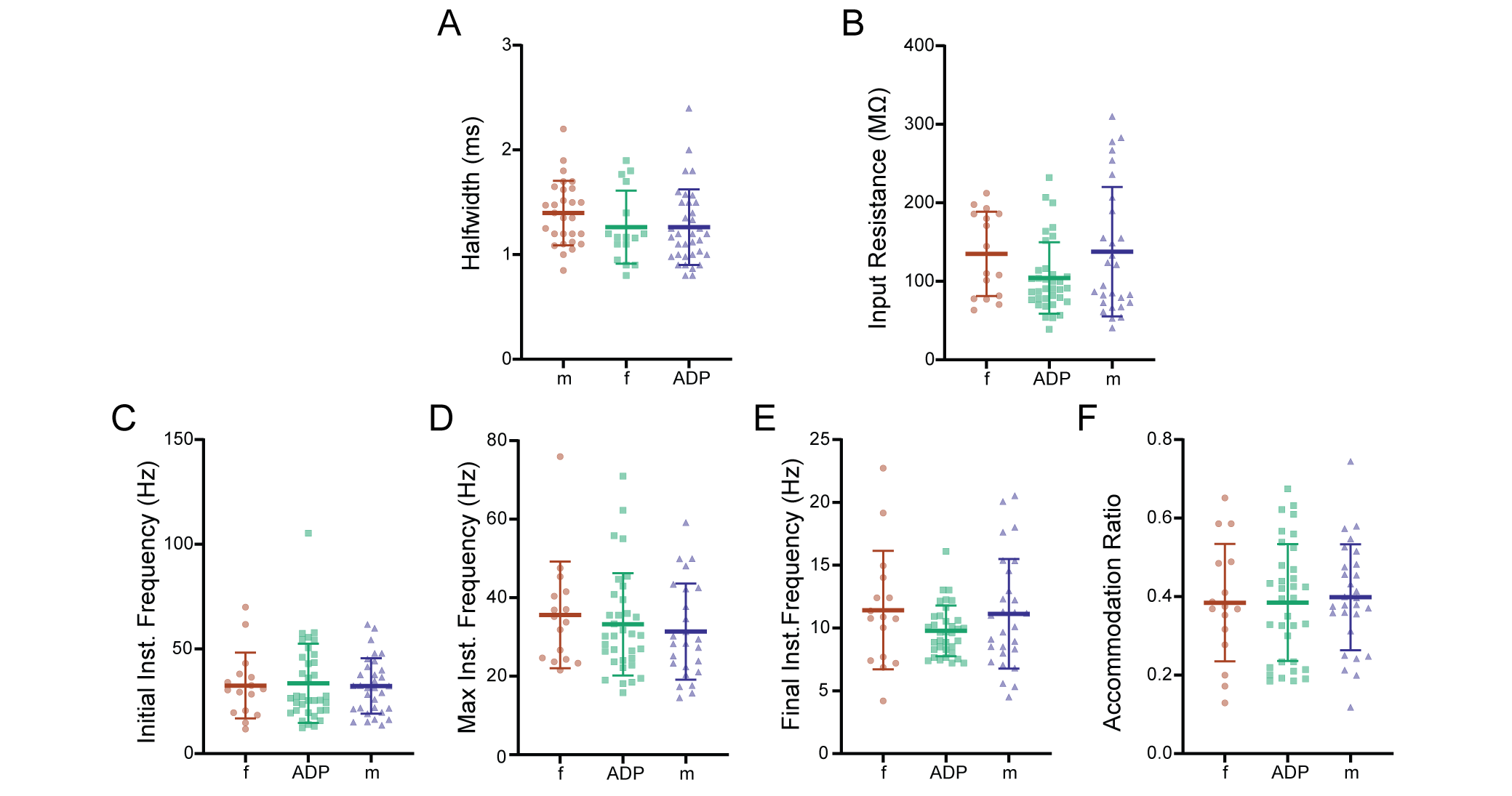

Supplement: Figure 3-1 — Other intrinsic properties of L2/3 PN subtypes based on AHP shape. Other analyzed intrinsic properties were graphed to show spread and variability as follows: A) AP half-width, B) input resistance, C) initial instantaneous frequency, D) max instantaneous frequency, E) final instantaneous frequency, and F) FR accommodation ratio. Scatter plots include mean values ± SD. Download Figure 3-1, TIF file. [file eneuro-12-ENEURO.0247-24.2025-s004.tif]
