# Supplementary material for: Characterizing the Diversity of Layer 2/3 Human Neocortical Neurons in Pediatric Epilepsy
Source: eNeuro. 2025 May 2;12(5):ENEURO.0247-24.2025. doi: 10.1523/ENEURO.0247-24.2025 (PMC12061357; doi:10.1523/ENEURO.0247-24.2025)
Supplement: Table 2-1 — Post hoc test p-values for statistically significant properties based on putative L2/3 PN subtype. The correlated post-hoc test for comparing intrinsic properties from Table 2. Download Table 2-1, DOCX file. [file eneuro-12-ENEURO.0247-24.2025-s005.docx]

**Table 2-1: Post hoc test *p*-values for statistically significant properties based on putative L2/3 PN subtype.**

| Intrinsic Property | *Multiple comparisons adjusted (p-value)* |
| --- | --- |
| Resting membrane potential (mV) |  |
| Input resistance (MΩ) |  |
| Voltage sag (%) |  |
| Membrane decay (ms) | Accommodating vs Notch – *p* = 0.0190 |
| AP threshold (mV) |  |
| AP amplitude (mV) | Stutter vs accommodating – *p* = 0.0050  Stutter vs Notch – *p* = 0.0185  Stutter vs ES – *p* = 0.0137 |
| AP half-width (ms) | Accommodating vs Notch – *p* = 0.0097 |
| AHP magnitude (mV) |  |
| AHP latency (ms) | Notch vs RS – *p* < .0001  Notch vs ES – *p* = 0.0172  Notch vs Accommodating – *p* = 0.0077 |
| ΔAHP (mV) |  |
| AP broadening ratio |  |
| AP amplitude adaptation ratio |  |
| Initial instantaneous frequency (Hz) | Accommodating vs RS – *p* = 0.0783  Accommodating vs Stutter – *p* = 0.0105  ES vs RS – *p* = 0.0076  ES vs Stutter – *p* = 0.0015 |
| Maximum firing rate (Hz) |  |
| Final instantaneous frequency (Hz) |  |
| Accommodation ratio | Stutter vs ES – *p* = 0.0086 |
