# Supplementary material for: Characterizing the Diversity of Layer 2/3 Human Neocortical Neurons in Pediatric Epilepsy
Source: eNeuro. 2025 May 2;12(5):ENEURO.0247-24.2025. doi: 10.1523/ENEURO.0247-24.2025 (PMC12061357; doi:10.1523/ENEURO.0247-24.2025)
Supplement: Table 2-2 — Definitions of electrophysiologic parameters. How each intrinsic property was calculated based on the square 600ms current steps. Download Table 2-2, DOC file. [file eneuro-12-ENEURO.0247-24.2025-s006.doc]

**Table 2-2: Definitions of electrophysiologic parameters.**

| Parameter | Protocol/ current step used | Description |
| --- | --- | --- |
| Resting membrane potential (mV) | Ramped current injection | Mean RMP (I_hold_ = 0pA) during a 500ms baseline across all sweeps |
| Input Resistance (MΩ) | -100 to 100pA series of current steps | The slope of the best fit line of the I-V plot |
| Membrane Decay τ (ms) | -100pA current step sweep | Determined using a single exponential fit, f(t) = A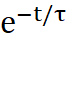to fit the change in voltage. |
| Voltage sag (%) | −200pA current step sweep | 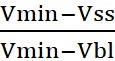x 100%. V_min_ - most hyperpolarized membrane voltage during the current injection, V_ss_ - mean steady-state membrane voltage (last 200ms of the current injection), V_bl_ - mean baseline membrane voltage (100ms before current injection) |
| AP threshold (mV) | Ramped current injection | The voltage at which dV/dT exceeded 20 V/s, calculated at the first AP |
| AP amplitude (mV) | Rheobase+100pA current step sweep | The voltage difference between threshold and AP peak |
| AP half-width (ms) | Rheobase current step sweep | The time between the half-amplitude point on the upslope of the AP waveform to the half-amplitude point on the downslope of the AP waveform |
| AHP magnitude (mV) | Rheobase current step sweep | The differences between the most hyperpolarized membrane voltage of the AHP (within 100ms after AP threshold) and AP threshold |
| AHP latency (ms) | Rheobase current step sweep | The time from AP threshold to the peak of the AHP. |
| ΔAHP | Rheobase current step sweep and the rheobase+100pA sweep | The difference between the first and last AHP magnitude (ΔAHP = AHP_last_– AHP_first_) |
| AP phase plot | Rheobase+100pA current step sweep | The rate of change of the mean AP as a function of the corresponding membrane voltage |
| AP broadening ratio | Rheobase current step sweep and the rheobase+100pA sweep | Broadening = half-width_second_/half-width_first_) |
| AP amplitude adaptation ratio | Rheobase current step sweep and the rheobase+100pA sweep | AP amplitude adaptation = mean amplitude_last 3 APs_/amplitude_first AP_ |
| Max Firing Rate (Collaborators) | Most depolarized current step before attenuation of FR | The number of action potentials (APs) over the length of the square current |
| FR ratio | All depolarizing current steps | Firing rate adaptation = ISI_first_/ISI_last ISI_. |
| Instantaneous frequency (Collaborators) | All depolarizing current steps | The initial and final instantaneous frequency plots were defined as 1/ISI_first_ and 1/ ISI_last_, respectively |
| Frequency (Collaborators) vs time (Avansini et al.) | All depolarizing current steps (1 sec long) | The number of action potentials over the length of the square current steps |
