# Supplementary material for: Characterizing the Diversity of Layer 2/3 Human Neocortical Neurons in Pediatric Epilepsy
Source: eNeuro. 2025 May 2;12(5):ENEURO.0247-24.2025. doi: 10.1523/ENEURO.0247-24.2025 (PMC12061357; doi:10.1523/ENEURO.0247-24.2025)
Supplement: Table 3-1 — Group comparison values with post hoc adjusted p-values for statistically significant properties based on AHP subtype. The correlated post-hoc test for comparing intrinsic properties from Table 3. Download Table 3-1, DOCX file. [file eneuro-12-ENEURO.0247-24.2025-s007.docx]

**Table 3-1: Group comparison values with post hoc adjusted *p*-values for statistically significant properties based on AHP subtype.**

| Intrinsic Property | (H, F, or W value, *p*-value) | Multiple comparisons adjusted  *(p*-value) |
| --- | --- | --- |
| Resting membrane potential (mV) | H(2) = 5.71, *p* = 0.0577 |  |
| Input resistance (MΩ) | H(2) = 2.75, *p* = 0.2531 |  |
| Voltage sag (%) | H(2) = 7.88, *p* = 0.0194 | fAHP vs mAHP – *p* = 0.0216 |
| Membrane decay (ms) | F = 0.34, *p* = 0.7114 |  |
| AP threshold (mV) | H(2) = 0.86, *p* = 0.6493 |  |
| AP amplitude (mV) | H(2) = 2.90, *p* = 0.2345 |  |
| AP half-width (ms) | H(2) = 3.97, *p* = 0.1375 |  |
| AHP magnitude (mV) | H(2) = 1.00, *p* = 0.6069 |  |
| AHP latency (ms) | H(2) = 53.57, *p* < 0.0001 | fAHP vs mAHP – *p* <0.0001  fAHP/ADP vs mAHP – *p* < 0.0001 |
| ΔAHP (mV) | H(2) = 16.15, *p* = 0.0003 | fAHP vs mAHP – *p* = 0.0066  fAHP/ADP vs mAHP – *p* = 0.0006 |
| AP broadening ratio | H(2) = 0.24, *p* = 0.8879 |  |
| AP amplitude adaptation ratio | H(2) = 11.90, *p* = 0.0026 | fAHP/ADP vs mAHP – *p* = 0.0017 |
| Initial instantaneous frequency (Hz) | H(2) = 0.12, *p* = 0.9414 |  |
| Maximum firing rate (Hz) | H(2) = 0.83, *p* =0.6588 |  |
| Final instantaneous frequency (Hz) | H(2) = 1.69, *p* = 0.4298 |  |
| Accommodation ratio | F = 0.08, *p* = 0.9230 |  |
| Max depolarization slope (dV/dT) | F = 2.12, *p* = 0.1265 |  |
| Max repolarization slope (dV/dT) | F = 5.28, *p* = 0.0072 | fAHP/ADP vs mAHP – *p* = 0.0049 |
