# Supplementary material for: Characterizing the Diversity of Layer 2/3 Human Neocortical Neurons in Pediatric Epilepsy
Source: eNeuro. 2025 May 2;12(5):ENEURO.0247-24.2025. doi: 10.1523/ENEURO.0247-24.2025 (PMC12061357; doi:10.1523/ENEURO.0247-24.2025)
Supplement: Table 4-1 — Group comparison values with post hoc adjusted p-values for statistically significant properties based on epilepsy subtype. The correlated post-hoc test for comparing intrinsic properties from Table 4. Download Table 4-1, DOCX file. [file eneuro-12-ENEURO.0247-24.2025-s008.docx]

**Table 4-1: Group comparison values with post hoc adjusted *p*-values for statistically significant properties based on epilepsy subtype.**

| Intrinsic Property | (H, F, or W value, *p*-value) | Multiple comparisons adjusted  *(p*-value) |
| --- | --- | --- |
| Resting membrane potential (mV) | F = 1.24, *p* = 0.2942 |  |
| Input resistance (MΩ) | H(2) = 9.97, *p* = 0.0068 | Control vs MCD – *p* = 0.0250  MCD vs OE – *p* = 0.0162 |
| Voltage sag (%) | H(2) = 8.58, *p* = 0.0137 | Control vs MCD – *p* = 0.0351  Control vs OE – *p* = 0.0241 |
| Membrane decay (ms) | H(2) = 0.16, *p* = 0.9238 |  |
| AP threshold (mV) | H(2) =28.23, *p* < 0.0001 | Control vs MCD – *p* < 0.0001  Control vs OE – *p* = 0.0002 |
| AP amplitude (mV) | H(2) = 24.56, *p* < 0.0001 | Control vs MCD – *p* = 0.0003  MCD vs OE – *p* < 0.0001 |
| AP half-width (ms) | H(2) = 10.10, *p* = 0.0064 | MCD vs OE – *p* = 0.0065 |
| AHP magnitude (mV) | F = 4.79, *p* = 0.0102 | Control vs MCD – *p* = 0.0118  Control vs OE – *p* = 0.0290 |
| AHP latency (ms) | H(2) = 4.95, *p* = 0.0843 |  |
| ΔAHP (mV) | H(2) = 0.2048, *p* = 0.9027 |  |
| AP broadening ratio | H(2) = 4.04, *p* = 0.1324 |  |
| AP amplitude adaptation ratio | W (DFn, DFd) = 3.35 (2.00, 56.20)  *p* = 0.0421 |  |
| Maximum firing rate (Hz) | F =6.49, *p* = 0.0022 | Control vs MCD – *p* = 0.0263  Control vs OE – *p* = 0.0016 |
| Accommodation ratio | W (DFn, DFd) = 3.52 (2.00, 58.54)  *p* = 0.0359 | MCD vs OE – *p* = 0.0339 |
| Max depolarization slope (dV/dT) | F = 5.54, *p* = 0.0051 | Control vs MCD – *p* = 0.0065  Control vs OE – *p* = 0.0241 |
| Max repolarization slope (dV/dT) | H(2) = 2.00, *p* = 0.3675 |  |
